# NAT10 promotes gallbladder cancer progression by remodeling cholesterol metabolism via PCSK9 mRNA acetylation

**DOI:** 10.1038/s41420-026-03104-z

**Published:** 2026-04-16

**Authors:** Zheng-yu Chen, Ming-yang Wang, Ben Ma, Cheng Zhao, Li-jia Pan, Zi-ying Wang, Yu-ting Wang, Pan-yi Mao, Xiang Zhao, De-long Qin, Yi-jun Shu, Yun-jiao Zhang, Shan-shan Xiang, Ping Dong

**Affiliations:** 1 Laboratory of General Surgery and Department of General Surgery, Xinhua Hospital affiliated with Shanghai Jiao Tong University School of Medicine, Shanghai, China; 2 Shanghai Key Laboratory of Biliary Tract Disease Research, China, Shanghai, China; 3 Department of Gastrointestinal Surgery, Shuguang Hospital, Shanghai University of Traditional Chinese Medicine, Shanghai, China; 4 Department of Cardio-Thoracic Surgery, Xinhua Hospital Affiliated to Shanghai Jiao Tong University School of Medicine, Shanghai, People’s Republic of China

**Keywords:** Cancer metabolism, Oncogenes

## Abstract

Gallbladder cancer (GBC) is a highly aggressive biliary tract tumor with a poor prognosis, underscoring the critical need for new therapeutic strategies. N-acetyltransferase 10 (NAT10), the sole writer of N4-acetylcytidine (ac4C), is upregulated in multiple cancers and is implicated in tumor pathogenesis. We observed significant NAT10 overexpression in GBC. Functional studies confirmed that NAT10 drives growth, migration, and malignant progression of GBC cells. We mechanistically linked this to NAT10-mediated ac4C modification, which stabilizes proprotein convertase subtilisin/kexin type 9 (PCSK9) mRNA, thereby reprogramming cholesterol metabolism and triggering intracellular cholesterol accumulation. This cholesterol buildup subsequently activates the PI3K/AKT pathway, stimulating cancer cell proliferation, migration, and invasion. Therapeutically, targeting NAT10 with Remodelin potently suppressed GBC proliferation. Importantly, Remodelin synergized with the standard chemotherapeutic agent gemcitabine to markedly enhance its therapeutic effect. Thus, our study defines a novel mechanism in which NAT10-dependent ac4C modification stabilizes PCSK9 mRNA to promote cholesterol-driven malignancy, nominating NAT10 as a compelling therapeutic target in GBC.

## Introduction

GBC is one of the most aggressive malignancies of the digestive system [1, 2]. Compared with other tumors, GBC is relatively rare; however, it is characterized by long latency and high invasiveness, resulting in a poorer prognosis [3]. Previous studies have identified several potential therapeutic targets, such as FOXO4 and PTBP3 [4, 5], and research on GBC treatment is ongoing [6, 7]. These findings offer new insights into GBC therapy. Although current treatments provide clinical benefit and improve prognosis in some patients, significant challenges remain. Consequently, the development of more effective and targeted therapeutic strategies remains necessary.

Beyond the genetic code, epigenetic RNA modifications represent a critical regulatory layer in cellular biology [8]. Their dysregulation is increasingly recognized as a contributor to diverse diseases, including cancer [9, 10]. By influencing key aspects of RNA biology, including synthesis, transport, stability, and translation efficiency, RNA modifications serve as fundamental mechanisms for regulating protein expression [11–13]. The oncogenic roles of several RNA modifications, including m6A, m7G, and m5C, have been extensively studied [14–16]. These post-transcriptional modifications form an extensive regulatory network for fine-tuning protein expressions, and elucidating this process is essential for understanding intracellular biological changes. Recently, ac4C modification has emerged as an important factor in cancer pathogenesis [17]. This modification occurs at cytosine residues in RNA and is present in both mRNA and tRNA. ac4C-modified mRNA exhibits altered stability and translation efficiency, which are critical for maintaining normal cellular function; however, aberrant modification contributes to disease development. NAT10 is an RNA acetyltransferase consisting of 1025 amino acids [18]. That catalyzes the formation of N4-acetylcytidine, an evolutionarily conserved modification present in both eukaryotes and prokaryotes [19]. One major mechanism of NAT10 action involves enhancing mRNA stability and translation efficiency through ac4C modification [20, 21]. Accordingly, NAT10 is highly expressed and functions as an oncogene in multiple cancer types [22, 23], underscoring its potential as a therapeutic target [24]. However, the role of NAT10 in GBC remains poorly understood.

Our study identifies NAT10 as a critical driver of GBC, with elevated expression correlating with poor patient prognosis. NAT10 promoted GBC progression both in vitro and in vivo. Integrated transcriptomic and metabolomic analyses revealed that NAT10 modulates cholesterol metabolism in GBC cells, with PCSK9 identified as a key downstream target. Mechanistically, NAT10 enhances PCSK9 mRNA stability, leading to increased PCSK9 expression and subsequent promotion of cholesterol synthesis. Cholesterol accumulation activates the PI3K/AKT signaling pathway, thereby promoting malignant GBC progression. In addition, the NAT10 inhibitor Remodelin suppressed tumor growth in vivo and in vitro. Remodelin synergized with gemcitabine to enhance its therapeutic effect. In summary, this study uncovers a new NAT10-driven mechanism regulating cholesterol metabolism, and identifies NAT10 as a promising therapeutic target for GBC.

## Results

### NAT10 is upregulated in gallbladder cancer and associated with poor prognosis

**Fig. 1:**
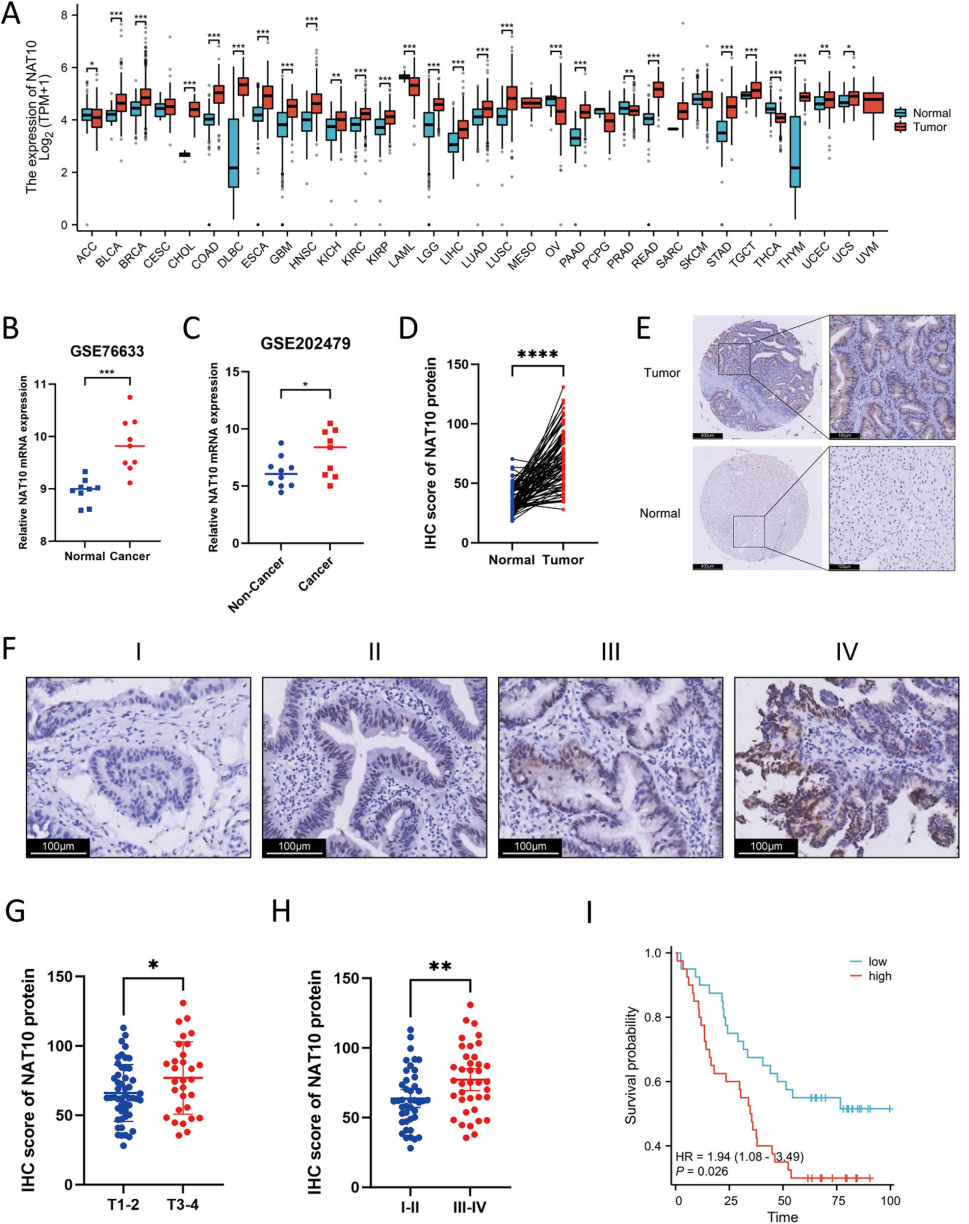
NAT10 is upregulated in gallbladder cancer and associated with poor prognosis. **A** Pan-cancer analysis of NAT10 expression in tumors versus adjacent normal tissues using TCGA data. **B**, **C** NAT10 mRNA expression in GBC versus paired adjacent tissues from the GSE76633 (**B**) and GSE202479 (**C**) datasets. **D** IHC scoring of NAT10 protein expression in 80 paired GBC and adjacent tissue specimens. **E** Representative IHC images of NAT10 staining in GBC and adjacent tissue. Scale bars: 100 μm and 400 μm. **F** Representative IHC images of NAT10 staining in GBC tissues at different clinical stages. Scale bar: 100 μm. **G**, **H** IHC scores of NAT10 stratified by T stage (**G**) and clinical stage (**H**). **I** Kaplan–Meier overall survival curves for GBC patients grouped by high or low NAT10 expression. Data are presented as mean ± SD. *p < 0.05, **p < 0.01, ***p < 0.001, ****p < 0.0001.

Analysis of the TCGA database revealed that NAT10 is frequently upregulated in multiple cancers (Fig. 1A), prompting us to investigate its role in GBC. Indeed, evaluation of two GEO datasets (GSE76633 and GSE202479) confirmed that NAT10 mRNA levels were significantly elevated in GBC tissues compared to adjacent non-tumor tissues and benign lesions (Fig. 1B, C) [25, 26]. This upregulation was further validated at the protein level using a tissue microarray (TMA) comprising 80 paired samples (Fig. 1D, E). Clinically, high NAT10 expression correlated with advanced T stage and overall clinical stage (Fig. 1F–H) and was associated with significantly shorter overall survival in patients (Fig. 1I). Collectively, our findings identify high NAT10 expression as a novel, independent prognostic biomarker in GBC.

### NAT10 promotes proliferation and migration in GBC

**Fig. 2:**
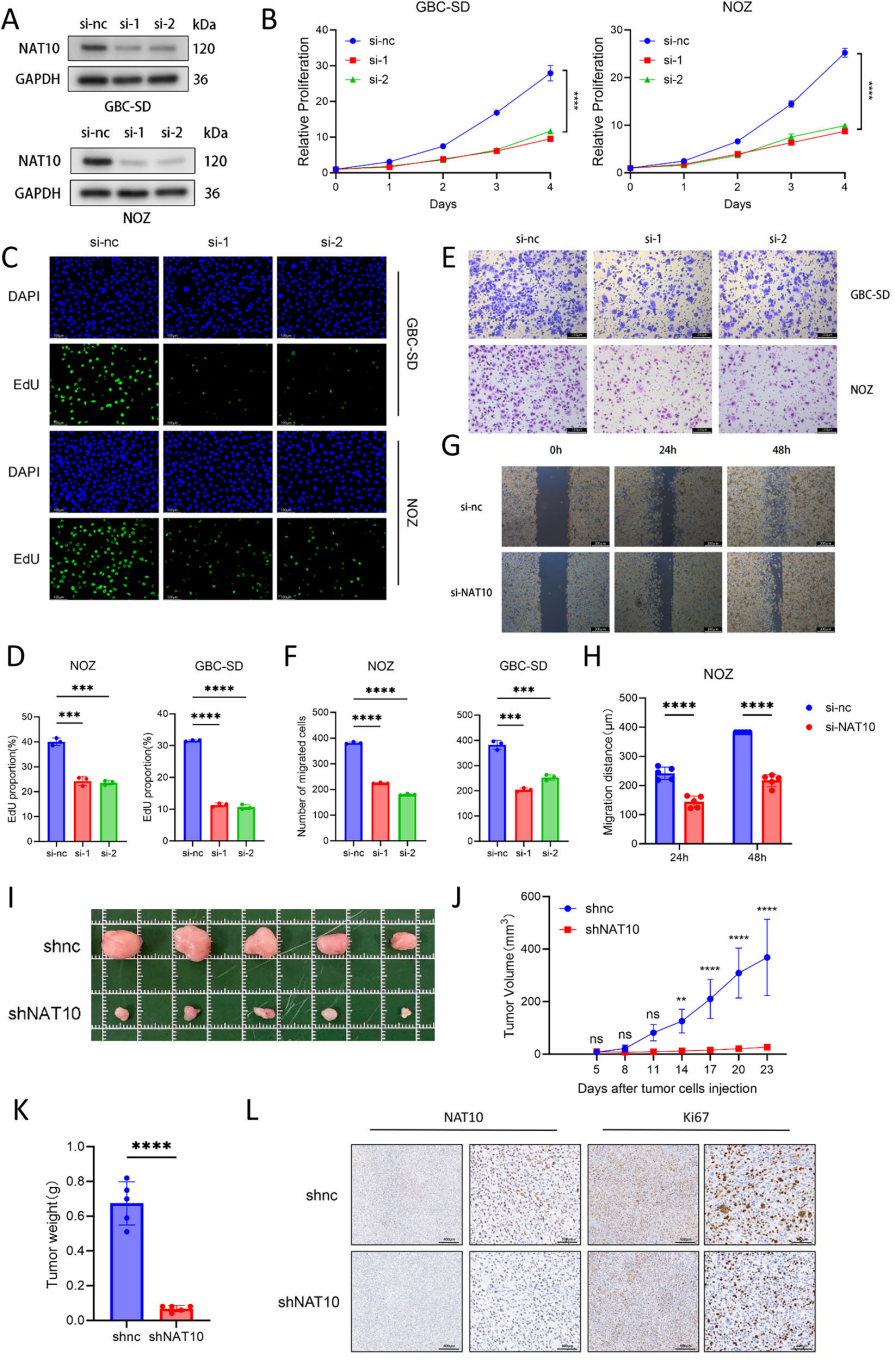
NAT10 promotes proliferation and migration in gallbladder cancer. **A** Western blot analysis confirming NAT10 knockdown efficiency. **B** Cell proliferation assessed by CCK-8 assay upon NAT10 knockdown. **C**, **D** EdU assay to evaluate proliferation in NAT10-knockdown cells. Scale bars: 100 μm. **E**, **F** Cell migration assessed by Transwell assay upon NAT10 knockdown. Scale bars: 200 μm. **G**, **H** Cell migration assessed by wound healing assay upon NAT10 knockdown. Scale bars: 100 μm. **I** Representative images of subcutaneous xenograft tumors from nude mice. **J**, **K** Tumor growth curves and final tumor weights of the xenografts. **L** Representative images of IHC staining for Ki-67 in tumor tissues. Scale bars: 100 μm and 400 μm. Data are presented as mean ± SD. *p < 0.05, **p < 0.01, ***p < 0.001, ****p < 0.0001.

To reveal the role of NAT10 in GBC, we selected the NOZ and GBC-SD cell lines, which exhibit relatively high NAT10 expression (Supplementary Fig. 1A), for further research. siRNA-mediated knockdown of NAT10 was performed, and its efficiency was confirmed by western blotting (Fig. 2A). Subsequent CCK-8 assays revealed that the proliferation of both NOZ and GBC-SD cells was significantly reduced following NAT10 knockdown (Fig. 2B). To further assess proliferation, EdU-488 labeling was used to evaluate DNA synthesis. The results indicated that DNA synthesis in NOZ and GBC-SD cells was markedly inhibited upon NAT10 knockdown (Fig. 2C, D). Transwell assays showed that the migratory ability of NOZ and GBC-SD cells was suppressed upon NAT10 knockdown (Fig. 2E, F). This finding was consistent with the results of the scratch assay, which also demonstrated a significant decrease in cell migration after NAT10 knockdown (Fig. 2G, H). Conversely, NAT10 overexpression enhanced the proliferation and migration abilities of NOZ and GBC-SD cells (Supplementary Fig. 1B–J). Collectively, these in vitro data demonstrate that NAT10 promotes GBC cell proliferation and migration.

Subsequently, to verify the role of NAT10 in vivo in GBC tumor formation, we established a stable NAT10-knockdown NOZ cell line and a control cell line (Supplementary Fig. 1K). In the subcutaneous tumor model of nude mice, tumor growth in the NAT10-knockdown group was suppressed compared to that in the control group (Fig. 2I–K). Furthermore, immunohistochemical (IHC) analysis of Ki67 revealed markedly lower scores in NAT10-knockdown tumors than in the controls (Fig. 2L). In conclusion, these results demonstrate that NAT10 promotes GBC proliferation in vivo.

### NAT10 promotes cholesterol metabolism reprogramming and activates the PI3K-AKT pathway in GBC

**Fig. 3:**
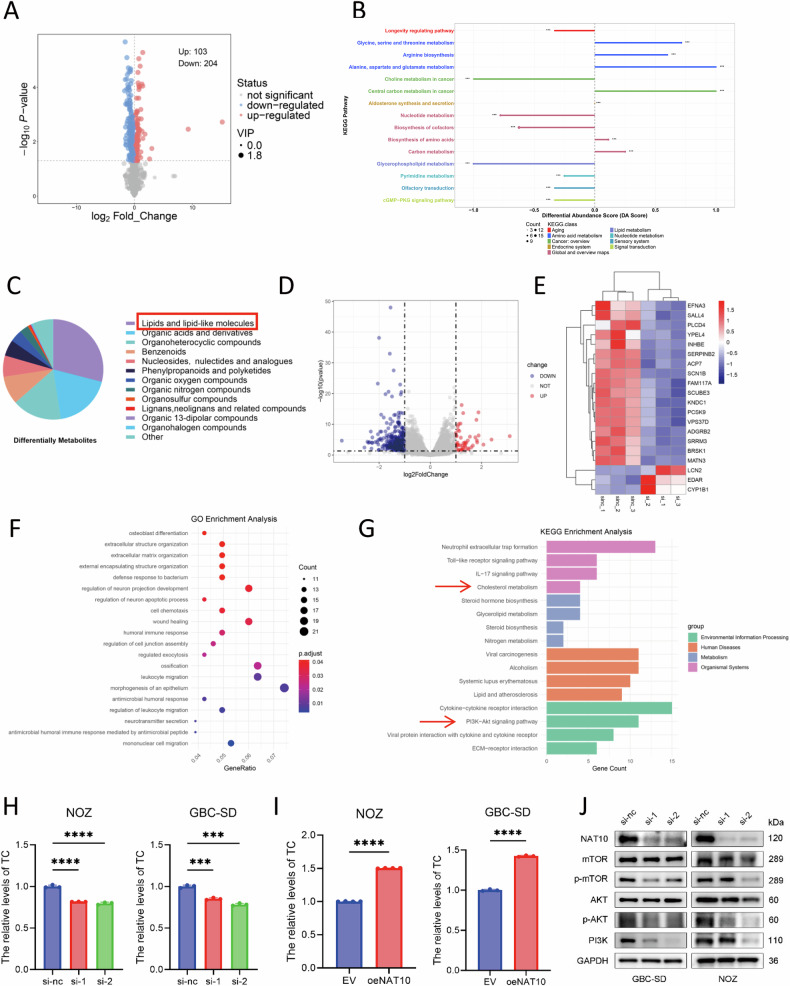
NAT10 promotes cholesterol metabolism reprogramming and activates the PI3K-AKT pathway in gallbladder cancer. **A** Volcano plot displaying differentially abundant metabolites between siNAT10 and control (siNC) NOZ cells. **B** KEGG pathway enrichment analysis of the differential metabolites. **C** Pie chart categorizing the types of differential metabolites identified upon NAT10 knockdown. **D** Volcano plot of DEGs between siNAT10 and siNC NOZ cells. **E** Heatmap of the top 20 most significantly altered DEGs after NAT10 knockdown. **F**, **G** GO and KEGG pathway enrichment analyses of the DEGs. **H**, **I** Relative total cholesterol levels following NAT10 knockdown or overexpression in NOZ and GBC-SD cells. **J** Western blot analysis of key PI3K/AKT/mTOR signaling components (PI3K, AKT, p-AKT, mTOR, p-mTOR) in NAT10-knockdown cells (GAPDH as loading control). Data are presented as mean ± SD. *p < 0.05, **p < 0.01, ***p < 0.001, ****p < 0.0001.

In most tumors, cancer cells reprogram their metabolism to support rapid proliferation [27–29]. To investigate the biological function of NAT10 in GBC metabolism, we performed metabolomic profiling of NOZ cell lines with NAT10 knockdown and control cells. Analysis of differential metabolites revealed 204 downregulated and 103 upregulated metabolites (Fig. 3A). KEGG enrichment and classification analyses indicated that after the knockdown of NAT10, the changes in lipid metabolism in GBC cells were the most significant (Fig. 3B, C).

To further elucidate the molecular mechanism underlying the changes in lipid metabolism, we conducted transcriptomic analysis of NAT10-knockdown and control NOZ cell lines. This identified 294 downregulated and 60 upregulated genes, from which the top 20 most significantly differentially expressed genes were selected (Fig. 3D, E). GO and KEGG enrichment analyses suggested that NAT10 knockdown modulates cholesterol metabolism in GBC cells (Fig. 3F, G). Given the established role of cholesterol in tumor progression [30–32], we hypothesized that NAT10 modulates cholesterol homeostasis. To verify this hypothesis, we first examined the effect of NAT10 on cholesterol levels. Intracellular cholesterol levels significantly decreased upon NAT10 knockdown in NOZ and GBC-SD cells and increased upon NAT10 overexpression (Fig. 3H, I). Next, we investigated the potential downstream consequences of cholesterol reprogramming. Our transcriptomic data suggested a potential link to the PI3K/AKT signaling pathway. Subsequently, western blot analysis confirmed that NAT10 knockdown suppressed PI3K/AKT pathway activation in NOZ and GBC-SD cells (Fig. 3J). These results indicate that NAT10 promotes intracellular cholesterol accumulation and activates the PI3K/AKT signaling pathway.

### NAT10 promotes cholesterol synthesis and uptake in GBC

**Fig. 4:**
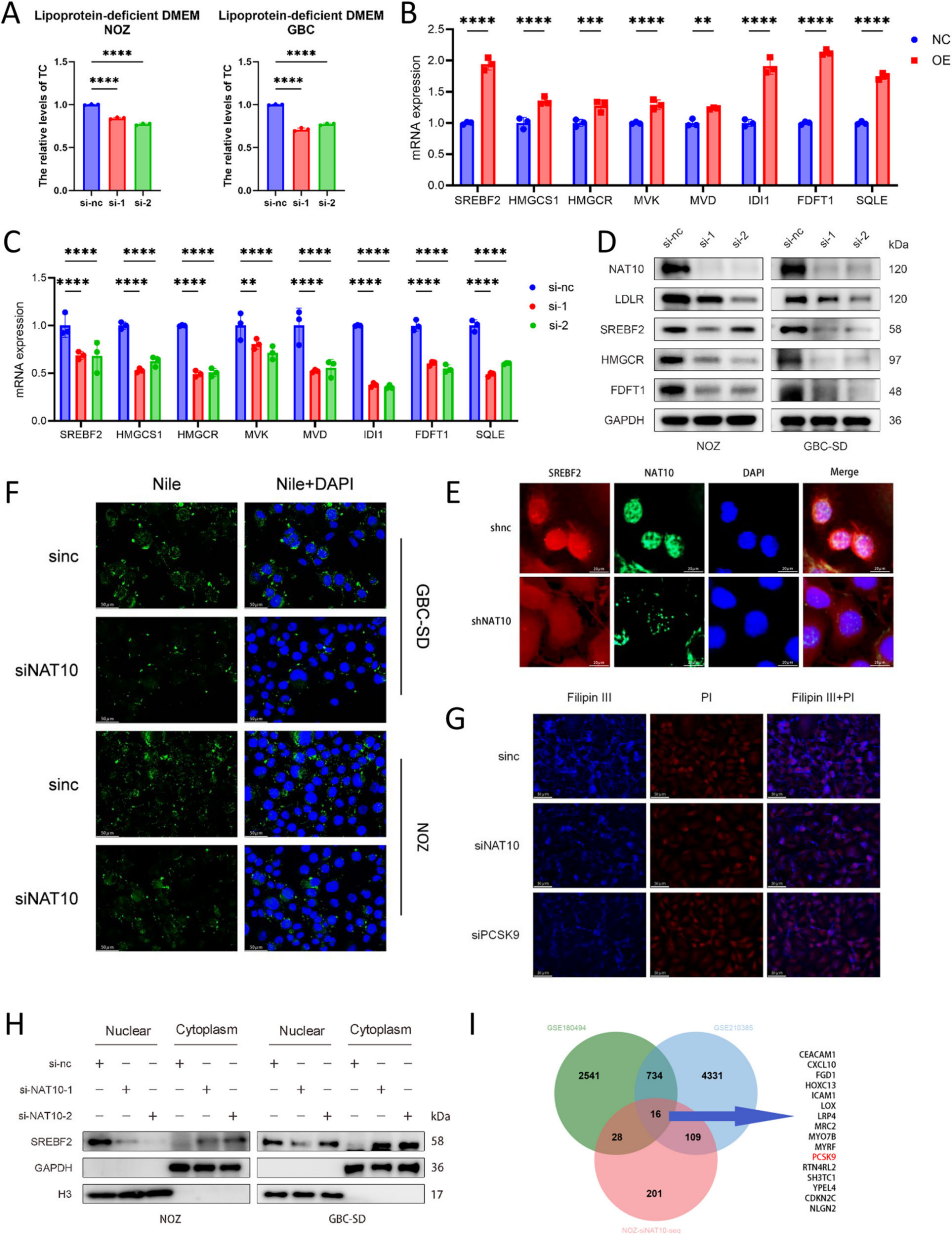
NAT10 promotes cholesterol synthesis and uptake in gallbladder cancer. **A** Relative total cholesterol levels in NAT10-knockdown cells cultured under lipoprotein-deficient (LPDS) conditions. **B**, **C** mRNA levels of cholesterol synthesis-related genes following NAT10 overexpression (**B**) or knockdown (**C**). **D** Western blot analysis of key cholesterol regulatory proteins (LDLR, SREBF2, HMGCR, FDFT1) in NAT10-knockdown NOZ and GBC-SD cells (GAPDH as loading control). **E** IF analysis of SREBF2 subcellular localization after NAT10 knockdown. Scale bar: 20 μm. **F** Lipid droplet staining using Nile Red in NAT10-knockdown cells. Scale bar: 50 μm. **G** Free cholesterol detection by Filipin III staining in NAT10- or PCSK9-knockdown cells. Scale bar: 50 μm. **H** Western blot analysis of SREBF2 expression in nuclear and cytoplasmic fractions after NAT10 knockdown. **I** Venn diagram showing the intersection (16 genes) of genes from two GEO datasets. Data are presented as mean ± SD. *p < 0.05, **p < 0.01, ***p < 0.001, ****p < 0.0001.

Cellular cholesterol is primarily acquired through de novo synthesis and exogenous uptake [33]. Following the integration of transcriptomic data from GBC tissues in the GEO database (GSE138109, GSE139682, and GSE202479), samples were stratified into low, medium, and high groups based on NAT10 expression levels [34, 35]. A heatmap was subsequently generated to illustrate the expression patterns of Cholesterol-related genes (CRGs), including LDLR, SREBF2, HMGCS1, HMGCR, MVK, MVD, IDI1, FDFT1, and SQLE. In the medium and high groups, the expression of these genes was significantly elevated compared to that in the low expression group (Supplementary Fig. 2A) [36, 37]. Correlation analysis further revealed that, except for MVD, NAT10 expression was positively correlated with the expression of these genes (Supplementary Fig. 2B, C). To determine how NAT10 alters cholesterol metabolism in GBC cells, we first cultured GBC cells in a cholesterol-free medium to block the uptake of exogenous cholesterol. We then measured the cholesterol levels in NAT10-knockdown NOZ and GBC cell lines, along with their corresponding controls. The results showed that after the removal of exogenous cholesterol, the cholesterol content in NAT10-knockdown NOZ and GBC-SD cells significantly decreased (Fig. 4A), indicating that NAT10 affects cholesterol metabolism through mechanisms other than influencing cholesterol intake. Subsequently, we assessed the expression of CRGs, in NOZ cells with either knockdown or overexpression of NAT10. NAT10 knockdown led to a marked decrease in the mRNA levels of CRGs, whereas NAT10 overexpression significantly increased CRGs (Fig. 4B, C). We next examined the protein expression of HMGCR, FDFT1, and SREBF2 using western blotting in NAT10-knockdown NOZ and GBC-SD cells. Consistent with the mRNA data, the protein levels of these genes were also reduced (Fig. 4D). SREBF2, a central transcription factor in cholesterol metabolism, requires nuclear localization to function [38]. NAT10 knockdown not only lowered the total SREBF2 protein (Fig. 4D) but also severely depleted its functional form in the nucleus. Fluorescence staining and nuclear-cytoplasmic fractionation experiments showed that the remaining SREBF2 was predominantly trapped in the cytoplasm (Fig. 4E, H), which explains the sharp decline in nuclear SREBF2. This indicates that NAT10 activates the entire synthetic program through a dual mechanism: controlling the expression of SREBF2 and promoting its nuclear translocation. In addition to de novo synthesis, exogenous uptake represents another major source of cholesterol [38]. NAT10 knockdown significantly reduced the expression of the LDL receptor (LDLR) responsible for endocytosing cholesterol [39](Fig. 4D). Accordingly, the combined inhibition of synthesis and uptake led to a substantial depletion of cellular cholesterol. Nile red staining revealed that the size and quantity of lipid droplets, which constitute the cholesterol reservoir [38], were significantly reduced (Fig. 4F). In addition to stored cholesterol, free cholesterol in the plasma membrane also contributes to cholesterol homeostasis. Filipin III staining revealed a significant decrease in membrane-free cholesterol following NAT10 knockdown (Fig. 4G). In conclusion, these findings establish NAT10 as a master regulator of cholesterol metabolism in GBC, coordinating both synthesis and uptake pathways.

### NAT10 affects cholesterol metabolism in GBC through the PCSK9-SREBF2 axis

**Fig. 5:**
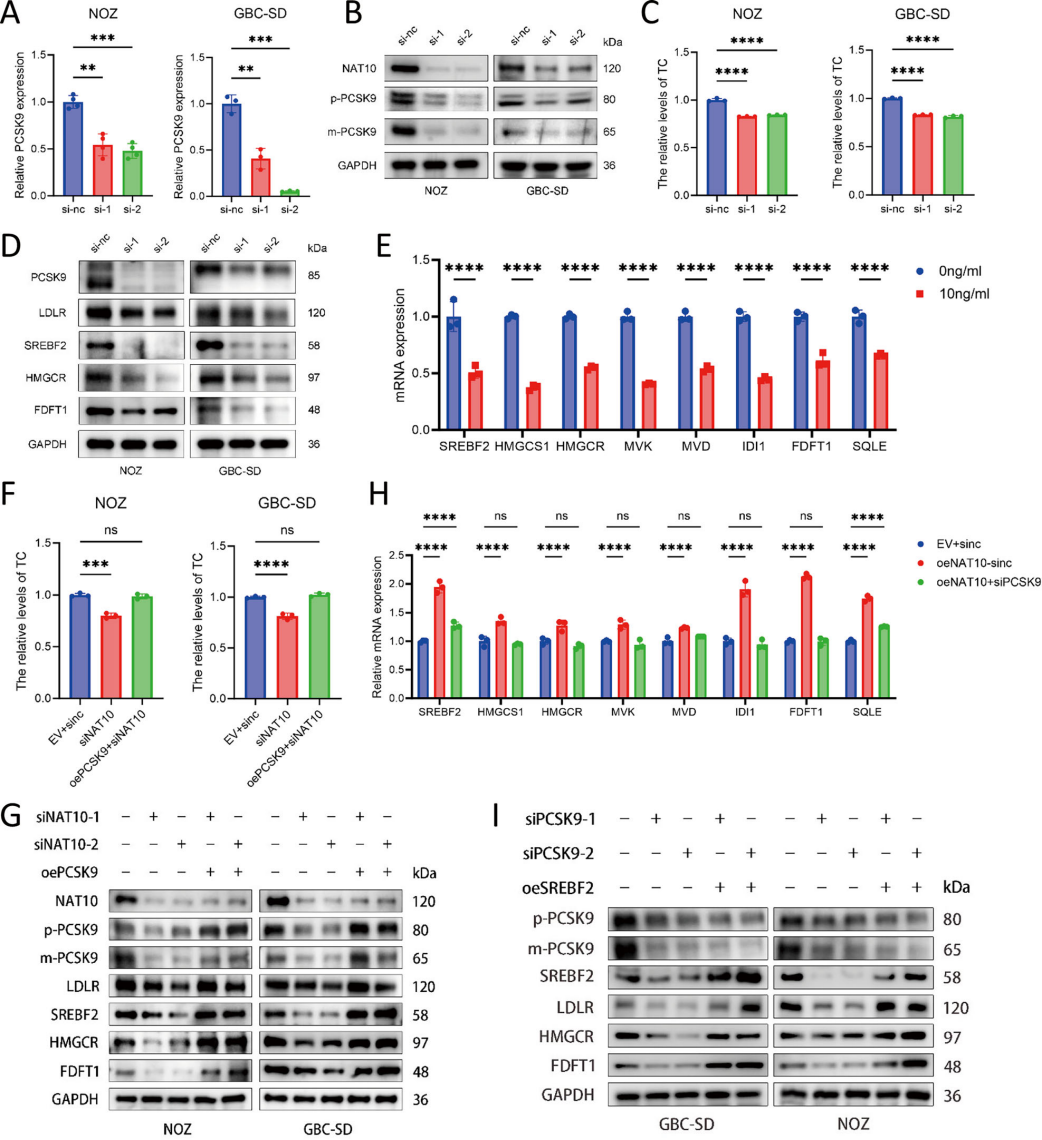
NAT10 affects cholesterol metabolism in gallbladder cancer through PCSK9-SREBF2 axis. **A** Relative PCSK9 mRNA levels upon NAT10 knockdown. **B** Western blot analysis of PCSK9 protein (with GAPDH loading control) in NOZ and GBC-SD cells after NAT10 knockdown. **C** Relative total cholesterol levels after PCSK9 knockdown. **D** Western blot analysis of key cholesterol metabolism regulators (LDLR, SREBF2, HMGCR, FDFT1; GAPDH as loading control) after PCSK9 knockdown in NOZ and GBC-SD cells. **E** mRNA levels of cholesterol synthesis-related genes after treatment with a PCSK9-IN-11 (10 ng/mL). **F** Relative cholesterol levels upon restoration of PCSK9 expression in NAT10-knockdown cells. **G** Western blot analysis of NAT10, PCSK9, and cholesterol metabolism regulators after PCSK9 restoration in NAT10-knockdown cells. **H** mRNA levels of cholesterol synthesis-related genes after PCSK9 restoration in NAT10-knockdown cells. **I** Western blot analysis of PCSK9 and cholesterol metabolism regulators after SREBF2 restoration in PCSK9-knockdown cells. Data are presented as mean ± SD. *p < 0.05, **p < 0.01, ***p < 0.001, ****p < 0.0001.

However, the mechanism by which NAT10 modulates cholesterol metabolism in GBC cells remains unclear. Based on its known role in mRNA acetylation, we hypothesized that NAT10 reprograms cholesterol metabolism by acetylating downstream targets [17]. To test this, we intersected genes identified from two GEO datasets of acRIP-seq (GSE180494 and GSE210385) after NAT10 knockdown with previously obtained transcriptomic differentially expressed genes, yielding 16 candidate genes [40, 41] (Fig. 4I). Among them, we focused on PCSK9 because it is a known positive regulator of cholesterol metabolism [42] and has been implicated in the progression of several tumors [43–45]. Analysis of transcriptomic data from GBC patients (GSE138109, GSE139682, and GSE2024279) revealed that PCSK9 expression was elevated in tumor tissues compared to adjacent non-tumor tissues and showed a positive correlation with NAT10 expression (Supplementary Fig. 2D, E). Therefore, we identified PCSK9 as a potential downstream effector of NAT10 in promoting cholesterol metabolism in GBC cells. We first validated that NAT10 knockdown significantly reduced PCSK9 expression in NOZ and GBC-SD cells (Fig. 5A, B). Functionally, knocking down PCSK9 (Supplementary Fig. 3A, B) recapitulated the NAT10-knockdown phenotype: it reduced intracellular cholesterol (Fig. 5C), decreased free cholesterol (Fig. 4G), and downregulated the protein levels of SREBF2, HMGCR, and FDFT1 (Fig. 5D). Similarly, PCSK9-IN-11, a PCSK9 inhibitor, suppressed the mRNA expression of SREBF2 and other synthesis genes (Fig. 5E). To ascertain whether PCSK9 was the primary downstream mediator, we performed rescue experiments. Crucially, overexpression of PCSK9 in NAT10 knockdown cells successfully rescued intracellular cholesterol levels (Fig. 5F) and restored the protein levels of SREBF2, HMGCR, FDFT1, and LDLR (Fig. 5G). Conversely, in NAT10 overexpressing cells, knocking down PCSK9 largely reversed the upregulation of cholesterol synthesis genes (Fig. 5H). These complementary results firmly established PCSK9 as a key effector downstream of NAT10. We then investigated how PCSK9 regulates cholesterol synthesis. Since SREBF2 is the master transcription factor [37, 46, 47], we tested whether it mediates PCSK9’s effects. Restoring SREBF2 expression in PCSK9 knockdown cells completely rescued the expression of downstream proteins, including LDLR, HMGCR, and FDFT1 (Fig. 5I). Taken together, our results delineate a clear signaling axis whereby NAT10 promotes cholesterol metabolism in GBC cells by regulating PCSK9, which in turn modulates the expression of SREBF2 and its downstream pathway.

### NAT10 promotes the proliferation and migration of GBC by upregulating PCSK9 via enhanced mRNA stability

**Fig. 6:**
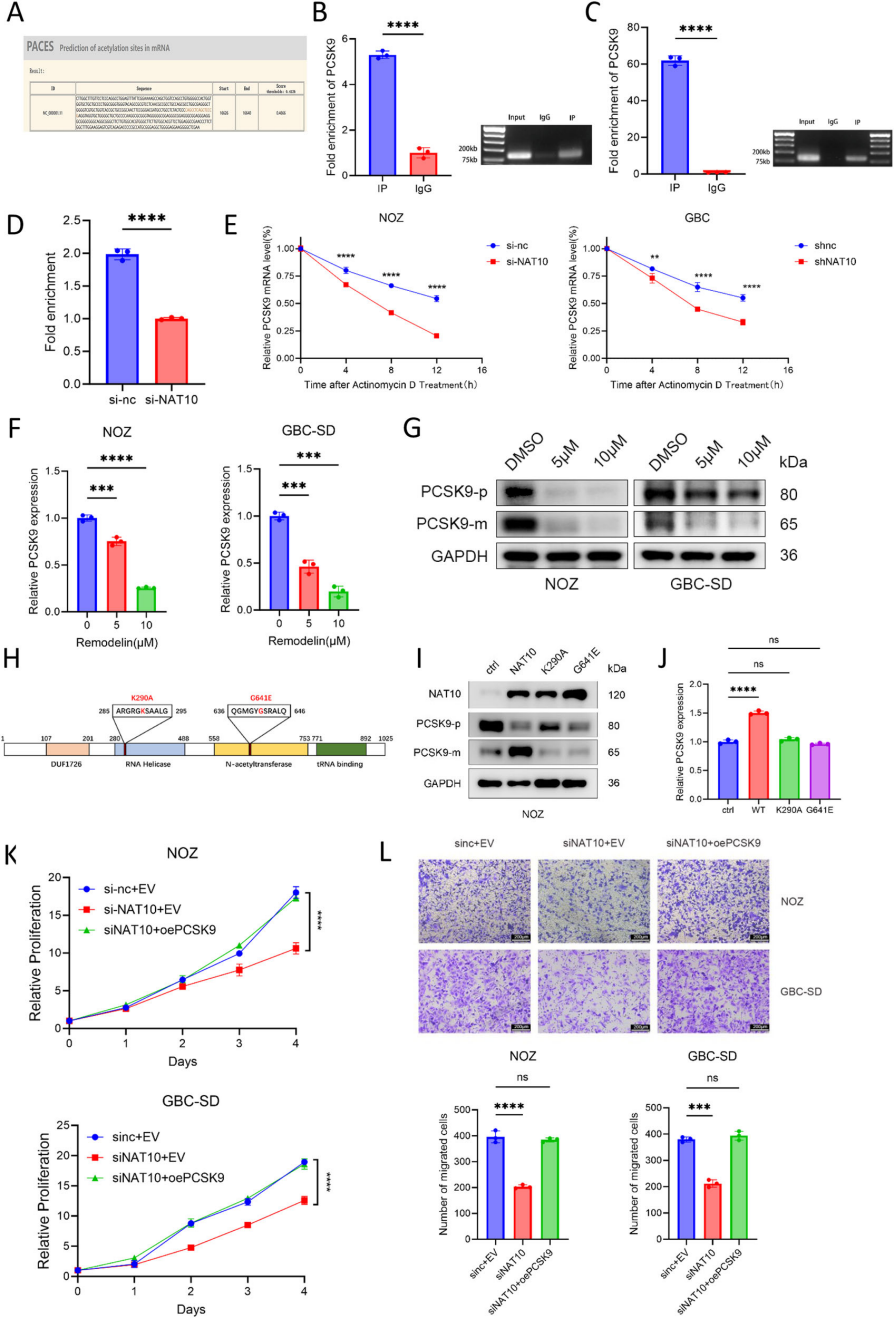
NAT10 promotes the proliferation and migration of gallbladder cancer by upregulating PCSK9 via enhanced mRNA stability. **A** Schematic of predicted acetylation sites on PCSK9 mRNA from an online prediction tool. **B**, **C** Validation of the NAT10-PCSK9 mRNA interaction. RNA immunoprecipitation (RIP) was performed using antibodies against NAT10 (**B**) or ac4C (**C**), followed by qPCR (left panels) and conventional PCR with gel electrophoresis (right panels) for PCSK9. **D** RIP-qPCR using anti-ac4C antibody in control (siNC) and NAT10-knockdown (siNAT10) NOZ cells. **E** PCSK9 mRNA stability assay using actinomycin D (10 ng/mL) in NAT10-knockdown cells. **F**, **G** Effect of the NAT10 inhibitor Remodelin (5, 10 μM) on PCSK9 expression at the mRNA (**F**) and protein (**G**) levels. GAPDH served as a loading control. **H** Schematic of NAT10 domain mutants: K290A in the RNA helicase domain and G641E in the N-acetyltransferase domain. **I**, **J** Effect of NAT10 wild-type (wt) and mutants (K290A, G641E) on PCSK9 expression at the protein (**I**) and mRNA (**J**) levels in NOZ and GBC-SD cells transfected with Flag-tagged constructs. **K**, **L** Rescue experiments: cell proliferation (**K**) and migration (**L**) were assessed by CCK-8 and Transwell assays, respectively, after restoring PCSK9 expression in NAT10-knockdown cells. Scale bar: 200 μm. Data are presented as mean ± SD. *p < 0.05, **p < 0.01, ***p < 0.001, ****p < 0.0001.

The mechanism by which NAT10 regulates PCSK9 expression remains unclear. Given the known function of NAT10, we hypothesized that NAT10 enhances the stability of PCSK9 mRNA via acetylation, thereby promoting its expression. Bioinformatic prediction using an mRNA acetylation database (http://rnanut.net/paces/) identified a high-scoring potential acetylation site within the region 16626-16640 of PCSK9 mRNA (Fig. 6A). To test whether NAT10 binds to PCSK9 mRNA, we performed RNA immunoprecipitation (RIP) using an NAT10 antibody. The results showed a significantly greater enrichment of PCSK9 mRNA in the NAT10 group than in the IgG control group, confirming a direct interaction (Fig. 6B). Furthermore, RIP using an anti-acetylated mRNA antibody also revealed strong enrichment of PCSK9 mRNA, indicating that PCSK9 mRNA is indeed acetylated (Fig. 6C). Importantly, NAT10 knockdown in NOZ cells markedly reduced the acetylation level of PCSK9 mRNA (Fig. 6D). Since NAT10 has been reported to stabilize target mRNAs through acetylation, we assessed PCSK9 mRNA stability in control and NAT10 knockdown NOZ and GBC-SD cells following transcriptional inhibition with actinomycin D. Following NAT10 knockdown, the half-life of PCSK9 mRNA was significantly shortened, supporting a role for NAT10 in stabilizing PCSK9 mRNA (Fig. 6E). Consistent with this finding, treatment of NOZ and GBC-SD cells with Remodelin, a specific NAT10 inhibitor [48], reduced the expression of both PCSK9 mRNA and protein in a dose-dependent manner (Fig. 6F, G). To directly test whether this regulation depends on NAT10’s acetyltransferase activity, we utilized mutants targeting its key functional domains: the K290A mutation in the RNA helicase domain and the G641E mutation in the N-acetyltransferase domain, both of which abrogate acetylation (Fig. 6H) [48–50] When expressed at comparable levels in NOZ cells, wild-type NAT10 increased PCSK9 protein expression, whereas the K290A and G641E mutants did not (Fig. 6I, J). This demonstrates that the acetyltransferase activity of NAT10 is essential for the upregulation of PCSK9. Collectively, our data establish that NAT10 binds to and adds ac4C to PCSK9 mRNA, enhancing its stability to promote PCSK9 protein synthesis.

Having established that NAT10 enhances PCSK9 expression by increasing the stability of PCSK9 mRNA, we investigated the functional role of PCSK9 in GBC. Given the role of PCSK9 in cholesterol metabolism and its emerging implications in cancer, we hypothesized that PCSK9 upregulation contributes to the malignant progression of GBC. To test this, we knocked down PCSK9 in NOZ and GBC-SD cell lines using siRNA. Functional assays revealed that PCSK9 knockdown significantly impaired the proliferation of GBC cells, as measured by CCK-8 and EdU assays (Supplementary Fig. 3C–E). Similarly, Transwell and scratch wound-healing assays showed a marked reduction in cell migration upon PCSK9 depletion (Supplementary Fig. 3F–I). To determine whether PCSK9 mediates the oncogenic effects of NAT10, we performed a rescue experiment by re-expressing PCSK9 in NAT10 knockdown NOZ and GBC-SD cells. Restoration of PCSK9 expression effectively rescued the impaired proliferation and migration abilities caused by NAT10 knockdown, as confirmed by CCK-8 and Transwell assays (Fig. 6K, L). As previous studies have provided evidence that PCSK9 regulates the PI3K/AKT signaling pathway [43, 51], we examined the status of this pathway in PCSK9 knockdown NOZ and GBC-SD. Our results demonstrated that PI3K/AKT signaling was significantly inhibited following PCSK9 knockdown (Supplementary Fig. 4A). Furthermore, when PCSK9 was re-expressed in NAT10 knockdown cells, the PI3K/AKT pathway was restored (Supplementary Fig. 4B). Collectively, these findings demonstrate that PCSK9 is a critical downstream effector of NAT10. NAT10 promotes the malignant progression of GBC likely through the PCSK9-mediated activation of the PI3K/AKT pathway, achieved by enhancing PCSK9 mRNA stability.

### NAT10 drives GBC progression by upregulating cellular cholesterol to activate the PI3K-AKT pathway

**Fig. 7:**
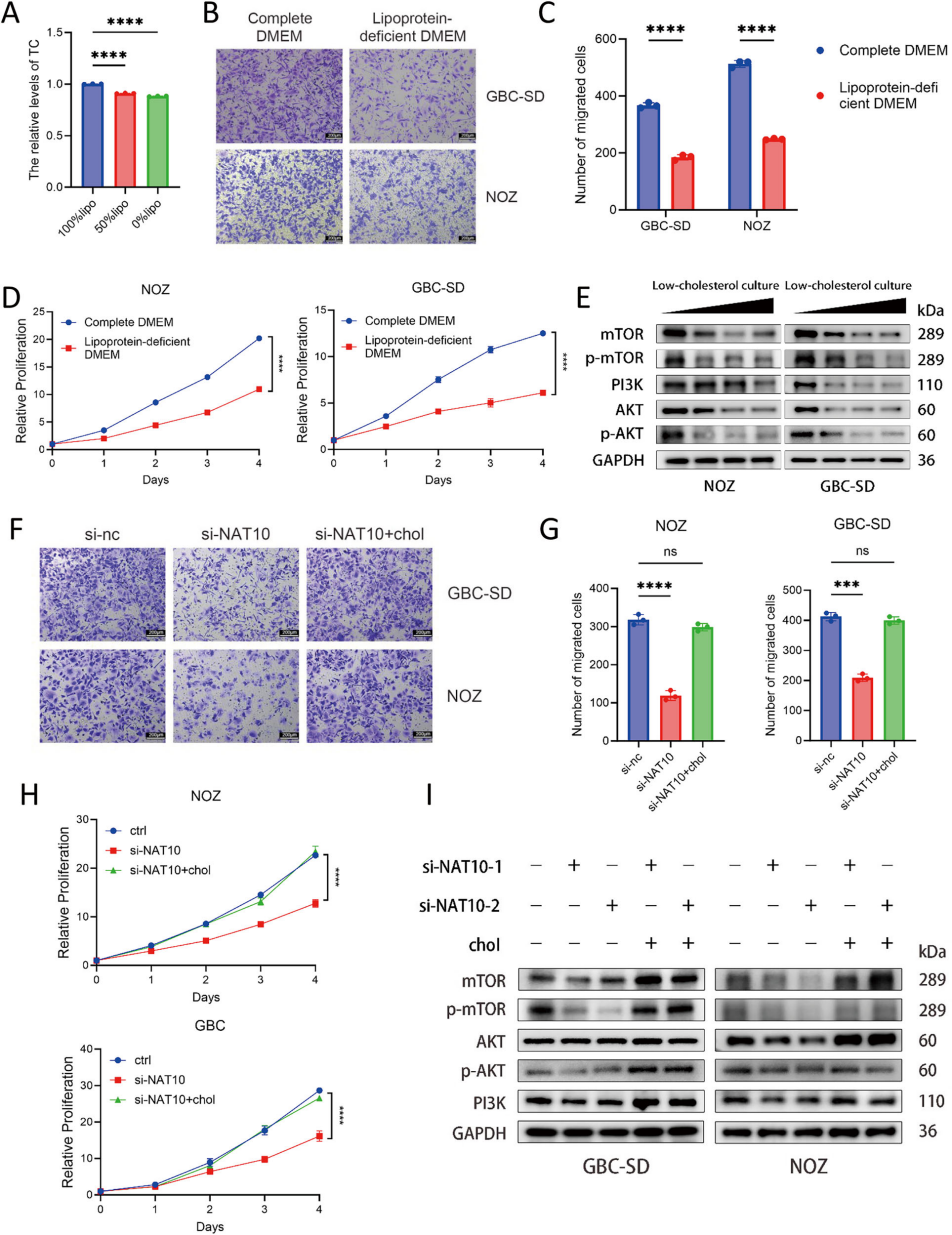
NAT10 drives gallbladder cancer progression by upregulating cellular cholesterol to activate the PI3K-AKT. **A** Relative total cholesterol levels in cells treated with the indicated concentrations of lipoprotein. **B**, **C** Cell migration assessed by Transwell assay following culture in complete or lipoprotein-deficient (LPDS) DMEM. Scale bars: 200 μm. **D** Cell proliferation measured by CCK-8 assay under complete or LPDS DMEM conditions. **E** Western blot analysis of mTOR, p-mTOR, AKT, p-AKT, and PI3K (with GAPDH as loading control) in cells cultured in LPDS DMEM. **F**, **G** Cell migration assessed by Transwell assay after cholesterol replenishment in NAT10-knockdown cells. Scale bars: 200 μm. **H** Cell proliferation measured by CCK-8 assay after cholesterol replenishment in NAT10-knockdown cells. **I** Western blot analysis of mTOR, p-mTOR, AKT, p-AKT, and PI3K after cholesterol replenishment in NAT10-knockdown cells. Data are presented as mean ± SD. *p < 0.05, **p < 0.01, ***p < 0.001, ****p < 0.0001.

Cholesterol plays diverse physiological roles in cells and is critically involved in tumor progression [31, 52]. Rapidly proliferating tumor cells require substantial amounts of cholesterol to support cell membrane assembly, as cholesterol is a key structural component [53, 54]. Moreover, many membrane receptors reside within lipid raft microdomains, which are largely composed of cholesterol [55, 56]. Thus, cholesterol metabolism influences cellular communication with the extracellular environment, and its dysregulation can lead to aberrant proliferation and chemotherapy resistance [57–60]. To investigate the biological function of cholesterol in GBC, we cultured NOZ and GBC-SD cells in lipid-depleted medium to achieve cholesterol deprivation. Using a cholesterol assay kit, we confirmed that intracellular cholesterol levels decreased in NOZ cells cultured in media with decreasing lipoprotein concentrations (Fig. 7A). We then assessed the functional consequences using Transwell and CCK-8 assays. Both migration and proliferation capabilities were significantly impaired in NOZ and GBC-SD cells under cholesterol-depleted conditions compared to those in complete medium (Fig. 7B–D). Furthermore, prolonged culture in lipid-depleted medium resulted in a marked inhibition of the PI3K/AKT signaling pathway in these cells (Fig. 7E). In NAT10 knockdown NOZ and GBC-SD cells, supplementation with exogenous cholesterol reversed the suppression of proliferation and migration caused by NAT10 loss (Fig. 7F–H). Consistent with this, cholesterol supplementation restored PI3K/AKT pathway activity, which was suppressed upon NAT10 knockdown (Fig. 7I). Collectively, these results demonstrate that the oncogenic effects of NAT10 are mediated through the maintenance of cellular cholesterol levels, which in turn activate the PI3K/AKT pathway to promote the proliferation and migration of GBC cells.

### Remodelin inhibits the proliferation of GBC and enhances the Gemcitabine chemotherapy in vitro and in vivo

**Fig. 8:**
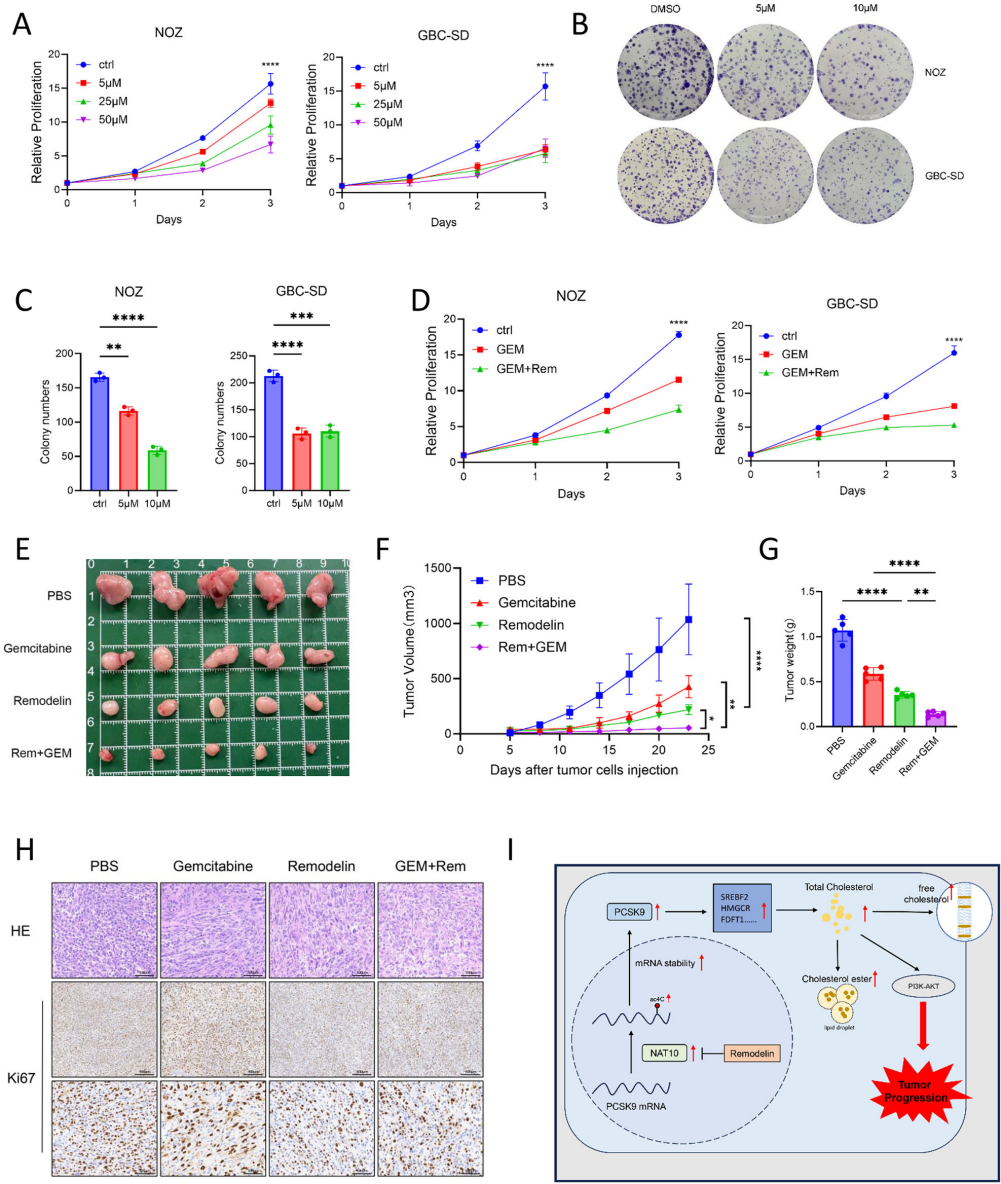
Remodelin inhibits the proliferation of gallbladder cancer and enhances the sensitivity to Gemcitabine chemotherapy in vitro and in vivo. **A** Cell viability measured by CCK-8 assay after treatment with Remodelin at indicated concentrations (0, 5, 25, 50 μM). **B**, **C** Clonogenic ability assessed by colony formation assay following treatment with Remodelin (0, 5, 10 μM). **D** Cell viability measured by CCK-8 assay after treatment with DMSO, gemcitabine (Gem), or Gem combined with Remodelin. **E** Representative images of subcutaneous tumors from nude mice treated with DMSO, Gem, Remodelin, or the combination. **F**, **G** Tumor growth curves and final tumor weights of the xenografts. **H** Representative IHC images showing Ki-67 expression in tumor tissues. Scale bars: 100 μm and 400 μm. **I** Schematic diagram depicting the proposed mechanism: NAT10 promotes GBC malignant progression via the PCSK9-SREBF2 axis, which reprograms cholesterol metabolism and activates the PI3K-AKT signaling pathway. Data are presented as mean ± SD. *p < 0.05, **p < 0.01, ***p < 0.001, ****p < 0.0001.

In this study, we assessed the therapeutic potential of Remodelin in GBC. Treatment of NOZ and GBC-SD cells with increasing concentrations of Remodelin (5, 25, and 50 μM) resulted in a dose-dependent suppression of cell proliferation, as measured by the CCK-8 assay (Fig. 8A). Consistent with this, Remodelin treatment markedly inhibited colony formation (Fig. 8B, C). PCSK9-IN-11 significantly reduced cell proliferation and clonogenic capacity, mirroring the effects observed with Remodelin treatment (Supplementary Fig. 5A–C). We determined the half-maximal inhibitory concentration (IC₅₀) values for both compounds in representative GBC-SD, NOZ, and HIBEpic. The results revealed pronounced selectivity: the IC₅₀ values for both the PCSK9 agent and Remodelin were significantly lower in GBC cells compared to HIBEpic. This indicates that GBC cells are substantially more sensitive to these treatments, suggesting a potential therapeutic window (Supplementary Fig. 6A). To further investigate this selectivity in a more physiologically relevant long-term context, we performed a colony formation assay. Consistent with the IC₅₀ data, chronic exposure to these agents resulted in markedly greater suppression of colony formation in GBC cells than in HIBEpic (Supplementary Fig. 6B–C). The inhibition rate in HIBEpic was significantly lower, reinforcing the notion that the anti-proliferative effects are preferentially targeted toward GBC cells. These findings imply that interventions targeting PCSK9 and its associated pathways may offer a promising therapeutic avenue for GBC with reduced anticipated toxicity toward the normal bile duct epithelium (Supplementary Fig. 6D). Having established that NAT10 drives cholesterol metabolism to promote GBC progression, and given the poor chemotherapy response in this cancer, we hypothesized that targeting NAT10 with Remodelin could not only inhibit tumor growth but also enhance the effectiveness of chemotherapy [59, 61, 62]. We found that the combination of Remodelin with gemcitabine more strongly inhibited cell proliferation than gemcitabine alone (Fig. 8D). We extended these findings to a subcutaneous tumor model in nude mice. Remodelin treatment significantly inhibited tumor growth, resulting in markedly reduced tumor volume and weight compared with those in the control group. Moreover, combination therapy suppressed tumor growth more effectively than monotherapy (Fig. 8E–G). IHC staining for Ki67 in tumor tissues revealed significantly lower scores in all treatment groups (Remodelin alone, gemcitabine alone, and the combination) than in the control group, with the most pronounced reduction occurring in the combination group (Fig. 8H). Altogether, our study fully maps the path in GBC from NAT10-mediated mRNA acetylation to PCSK9-driven metabolic reprogramming. The potent antitumor response to Remodelin validates this entire axis as clinically relevant, transforming a fundamental epitranscriptomic discovery into a druggable therapeutic target against GBC (Fig. 8I).

## Discussion

NAT10 is an acetyltransferase that promotes tumor progression in multiple cancer types. Previous studies have shown that NAT10 stabilizes MDM2 in gastric cancer and regulates EGFR translation in esophageal cancer[40, 61]. However, the role of NAT10 in GBC remains unclear. In this study, NAT10 was identified as a key gene in GBC. Frequent overexpression in tumor tissues correlated with poor patient prognosis, and functional experiments confirmed its critical role in promoting GBC cell proliferation and migration. These findings support NAT10 as a potential prognostic biomarker and therapeutic target in this highly lethal malignancy. NAT10 also plays an important role in reshaping tumor energy metabolism. In osteosarcoma, NAT10 alters glycolysis through the YTHDC1- LDHA/PFKM axis[63], whereas in triple-negative breast cancer, it promotes glycolytic addiction through its acetylation function, thereby facilitating immune evasion [64]. Given the reported role of NAT10 in regulating glucose metabolism in cervical cancer [65], we investigated whether NAT10 exerts a similar metabolic function in GBC. Integrated transcriptomic and metabolomic analyses revealed a marked disruption of cholesterol metabolism following NAT10 knockdown. This finding is significant as cancer cells depend on altered cholesterol homeostasis to maintain membrane integrity, lipid raft formation, and signal transduction [38, 54]. Cholesterol has long been recognized as a key contributor to tumor initiation and progression. Inhibition of cholesterol synthesis enhances antitumor immunity in colon cancer [66], whereas excessive cholesterol synthesis in cervical cancer promotes activation of the PI3K/AKT signaling pathway [56]. In this study, cholesterol supplementation reversed the proliferative and migratory defects caused by NAT10 loss in GBC, firmly establishing cholesterol metabolism as a key downstream pathway. Emerging evidence suggests that reprogrammed cholesterol metabolism contributes to gemcitabine resistance [67, 68]. Although our study identifies NAT10 as a regulator of cholesterol biology, its specific role in conferring gemcitabine resistance remains unclear. The NAT10–PCSK9–SREBF2 axis is central to cholesterol metabolism in GBC. Mechanistically, NAT10 directly binds to and acetylates PCSK9 mRNA, thereby enhancing its stability. Elevated PCSK9 increases SREBF2 expression, which in turn upregulates its target genes, including LDLR, HMGCR, and FDFT1; however, the precise molecular mechanism by which PCSK9 regulates SREBF2 protein levels requires further investigation. PCSK9 is known to promote LDLR degradation [69], but we observed that this effect is overshadowed by the dominant regulation of LDLR by SREBF2. The role of PCSK9 in tumor immune responses also warrants attention. In liver cancer, the combination of PCSK9-neutralizing antibodies and PD-1 monotherapy has demonstrated potent antitumor effects, and PCSK9 is closely associated with immune escape [70, 71]. Future studies using immunocompetent models will therefore be necessary to elucidate how PCSK9 shapes the GBC immune microenvironment. PCSK9 is a critical mediator of the regulatory effect of NAT10 on the PI3K/AKT signaling pathway. Our study established a linear PCSK9–PI3K/AKT axis. However, the oncogenic network driven by NAT10 is likely more complex than this defined pathway. This complexity suggests that NAT10 may also regulate PI3K/AKT signaling through PCSK9-independent mechanisms or coordinate parallel tumor-promoting pathways. In vitro experiments using Remodelin and PCSK9 inhibition confirmed that GBC cells are highly sensitive to these treatments and exhibit reduced toxicity toward normal bile duct epithelial cells, indicating a promising therapeutic avenue for GBC.

In summary, NAT10 is upregulated in GBC and is associated with poor prognosis. NAT10 promotes GBC cell proliferation and migration by enhancing PCSK9 expression, thereby stimulating cholesterol synthesis. Mechanistically, NAT10 mediates N4-acetylcytidine modification of PCSK9 mRNA, increasing its stability and upregulates PCSK9 expression. Elevated PCSK9 activates the SREBF2 pathway, leading to increased expression of cholesterol-related genes, such as HMGCR and FDFT1, and enhanced cholesterol synthesis. Excessive cholesterol synthesis subsequently activates the PI3K/AKT signaling pathway, promoting GBC cell proliferation and migration. Furthermore, combining gemcitabine with the NAT10 inhibitor Remodelin improved the therapeutic efficacy of gemcitabine. These findings provide deeper insight into aberrant cholesterol metabolism during GBC progression and identify a novel therapeutic target for GBC treatment.

## Materials and methods

### Clinical samples and IHC

A GBC tissue microarray (TMA was obtained from Shanghai Mingyi Biotechnology (Shanghai, China; product code: LD-GAC1601). The TMA comprised 80 cases of gallbladder cancer and 80 cases of adjacent normal tissues.

For immunohistochemistry, tissue slides were rehydrated and incubated in 3% H_2_O_2_ for 5 min, boiled with 0.01 M sodium citrate buffer, and subjected to antigen recovery, followed by blocking with 5% goat serum. Subsequently, the slides were incubated with the primary antibody at 4 °C overnight. The next day, the slides were incubated with goat anti-rabbit secondary antibody conjugated to horseradish peroxidase for 1 h at room temperature. Subsequently, the sections were stained with 3,3’-diaminobenzidine and counterstained with hematoxylin Sigma. (For multiple immunohistochemistry, tissue slides were incubated with tyramine signal-amplification-conjugated fluorophores for 10 min at room temperature after IHC primary and secondary antibody staining. All conjugates other than fluorophores were removed from the tissue slides using an antigen recovery procedure, followed by primary antibody-secondary antibody-TSA-conjugated fluorophore staining. Three researchers independently assessed the immunostaining scores. Information on all antibodies used is listed in Supplementary Table 3.

### Cell culture and reagents

The human GBC cell lines GBC-SD, SGC-996, NOZ, OCUG-1, and EH-GB1, as well as the HEK293T cell line, were obtained from the following sources: GBC-SD and SGC-996 from the Shanghai Institute of Biological Sciences, Chinese Academy of Sciences (Shanghai, China); EH-GB1 from the Health Science Research Resources Bank (Osaka, Japan); OCUG-1 and NOZ from the Japanese Collection of Research Bioresources Cell Bank (JCRB). All cells were cultured in high-glucose DMEM (Gibco, USA) supplemented with 10% fetal bovine serum (FBS) and 1% penicillin-streptomycin at 37 °C in a humidified 5% CO₂ incubator. Remodelin\PCSK9-IN-11\Gemcitabine used in the study was purchased from MedChemExpress.

### RNA interference and lentiviral transduction

All siRNAs and NAT10-specific shRNAs were synthesized by Tsingke Biotechnology (Shanghai, China). All the overexpression plasmids were purchased from Miaoling Biological (Wuhan, China). The sequences of all siRNAs, NAT10-specific shRNA, and overexpression plasmids are listed in Supplementary Table 1.

For transient transfection, siRNAs were delivered into cells using the Rfect transfection reagent, according to the manufacturer’s instructions. For stable overexpression or knockdown, lentiviruses were produced by cotransfecting the target plasmids with packaging plasmids (plp1, plp2, and plp-vsvg) at a predetermined ratio. Lentivirus-containing supernatants were collected at 24, 48, and 72 h post-transfection, concentrated, and used to infect cells to establish stable cell lines.

### RNA extraction and quantitative real-time PCR

Total RNA was isolated using the EZ-press RNA Purification Kit (EZBioscience, USA). cDNA was generated in a reverse transcription using the HiScript II One Step RT-PCR Kit (Vazyme, China). Subsequent quantitative PCR amplification was performed using ChamQ SYBR qPCR Master Mix (Vazyme, China), following the manufacturer’s protocol. The primer sequences are listed in Supplementary Table 2.

### Western blot analysis

Proteins were separated by electrophoresis on a 4–20% gel (ACE Biotechnology, China) and transferred onto a PVDF membrane (Millipore, USA). The membrane was blocked with 5% skim milk and incubated overnight at 4 °C with primary antibodies at the recommended dilutions. After washing three times with TBST, the membrane was incubated with an HRP-conjugated secondary antibody (Abclonal, Wuhan, China) for 1 h at room temperature. Protein bands were detected using an enhanced chemiluminescence reagent (Meilunbio, China). All antibodies used are listed in Supplementary Table 3.

### Cell proliferation assay

Cell proliferation was assessed using the Cell Counting Kit-8 (CCK-8, Yeasen) and EdU-488 detection kit (Beyotime, China), according to the manufacturers’ instructions. For the colony formation assay, 1000 treated cells were seeded per well in six-well plates and cultured for 10 d. The resulting colonies were fixed with 4% paraformaldehyde, stained with 0.1% crystal violet, and rinsed with water. Colonies containing >50 cells were counted.

### Transwell migration assay

Cell migration was assessed using Transwell chambers (Corning, USA). The lower chamber was filled with 500 μL of medium containing 10% FBS as a chemoattractant. Then, 200 μL of serum-free medium containing 2 × 10⁴ cells was added to the upper chamber. After incubation for 24 h, non-migratory cells on the upper surface of the membrane were carefully removed using a cotton swab. Cells that migrated to the lower surface were fixed with 4% paraformaldehyde, stained with 0.1% crystal violet, and then imaged and counted under an inverted microscope.

### Wound healing assay

Cells were seeded in 6-well plates and cultured until they reached 90% confluency. A straight wound was created in the cell monolayer using a 200 μL pipette tip. Cell debris was gently washed away with phosphate-buffered saline (PBS), and the medium was replaced with serum-free medium. The cells were subsequently incubated in serum-free medium to minimize cell proliferation. Wound images at the same location were captured at 0, 24, and 48 h using an inverted microscope. Wound width was measured using ImageJ software, and the percentage of wound closure was calculated.

### Transcriptome and lipidome profiling

Transcriptomic (RNA-seq) and non-targeted lipid metabolomic analyses were performed by Tsingke Biotechnology (Shanghai, China). The RNA libraries were constructed on the Illumina NovaseqTM 6000 platform by Tsingke Biotechnology. Cell samples for RNA-seq were lysed using TRIzol (Invitrogen) and stored at −80 °C. The lipidomics samples were immediately flash-frozen in liquid nitrogen after collection. All samples were shipped to the vendor under appropriate cold conditions for processing at the laboratory. The RNA-seq data were analyzed using R, and the lipidomic data were processed using the vendor’s proprietary pipeline.

### RIP and acetylated RNA immunoprecipitation (acRIP)

The RIP assay was performed using a commercial kit (Bersinbio, China) according to the manufacturer’s instructions. The acRIP procedure was as follows: Total RNA was extracted from cells using TRIzol reagent (Invitrogen). The RNA was pre-cleared with protein A/G magnetic beads (10 μL per sample) for 1 h at 4 °C with rotation to remove nonspecifically binding species. The pre-cleared RNA was then immunoprecipitated by incubation with an anti-ac4C antibody (5 μg per sample; Abclonal) overnight at 4°C. Protein A/G magnetic beads (20 μL per sample) were added and incubated for an additional 1 h. After magnetic separation, the supernatant was removed, and the beads were washed three times with the immunoprecipitation buffer. Finally, the bound RNA was eluted using an RNA elution buffer.

### mRNA stability assay

To assess mRNA stability, NAT10-knockdown (sh-NAT10) and control (sh-nc) cells were treated with actinomycin D (5 μg/mL) to halt transcription. Cells were collected at the indicated time points (0, 4, 8, and 12 h). Total RNA was isolated using TRIzol reagent, and the relative abundance of the target mRNAs was determined using RT-qPCR.

### Immunofluorescence (IF) assay

After the cells adhered to the slides, they were fixed with 4% paraformaldehyde. Deparaffinized and rehydrated tissue sections were subjected to heat-induced antigen retrieval. After blocking with 5% normal serum, the sections were incubated overnight at 4°C with primary antibodies, followed by incubation with species-matched fluorophore-conjugated secondary antibodies for 1 h at room temperature. The cell nuclei were counterstained with DAPI. The slides were mounted with an anti-fade medium and imaged using a fluorescence microscope. The controls included the omission of the primary antibody. All steps, including staining and image acquisition, were performed by RecordBio (Shanghai).

### Nile red staining

Lipid droplets were stained using a Nile Red staining kit (Beyotime, China) following the manufacturer’s instructions.

### Filipin III staining

The cells were seeded in six-well plates. After fixation with 4% paraformaldehyde, the cells were stained with 0.1 mg/mL Filipin III (Absin, Shanghai, China) for 30 min at room temperature, followed by incubation with 0.3 μg/mL propidium iodide (PI; Beyotime, China) for 10 min. Fluorescence images were captured using an inverted fluorescence microscope.

### Cholesterol measurement

Cellular cholesterol levels were measured using the Amplex Red Cholesterol and Cholesteryl Ester Assay Kit (Beyotime, China) according to the manufacturer’s instructions.

### Half-maximal inhibitory concentration (IC₅₀)

Following 48 h of drug exposure, 10 µL of Cell Counting Kit-8 (CCK-8) reagent was added directly to each well. The plates were returned to the incubator for 2 h. The absorbance of each well was then measured at a wavelength of 450 nm using a microplate reader. The average absorbance of the blank wells was subtracted from all readings. Cell viability for each treatment group was expressed as a percentage relative to the vehicle control group (set as 100%). The dose-response data (drug concentration versus percentage of cell viability) were fitted using nonlinear regression analysis in GraphPad Prism software. The half-maximal inhibitory concentration (IC₅₀) was derived from the fitted curve as the drug concentration that reduced cell viability by 50% compared to the control.

### Xenograft model

BALB/c-nu Mice purchased from GemPharmatech Co., Ltd (Jiangsu, China). Subcutaneous tumor models were established in male nude mice. All animal procedures were approved by the Ethics Committee of Xinhua Hospital, Shanghai, China. The mice were randomly assigned to experimental or control groups. A suspension of 2 × 10⁶ cells in 100 μL of PBS was injected into the left axilla of each four-week-old nude mouse. Tumor volume was measured every third day of the experiment. One week after inoculation, the mice were randomly divided into four treatment groups. The groups received intraperitoneal injections every two days with one of the following: 100 μL normal saline (control), 2 mg/kg Remodelin, 50 mg/kg gemcitabine, or a combination of 2 mg/kg Remodelin and 50 mg/kg gemcitabine. All mice were euthanized at the appropriate endpoints.

### Statistical analysis

Statistical analyses and graph generation were performed using GraphPad Prism (10.1.2). For comparisons between the two groups, a two-tailed Student’s t-test was used to calculate the *P* values. Comparisons among more than two groups were conducted using analysis of variance. A *P* value <0.05 was considered statistically significant, with the following conventions: *P* < 0.05: *p < 0.01: **p < 0.001: ***p < 0.0001: ****. Univariate survival analysis was performed using the Kaplan–Meier method.

## Supplementary information

Supplementary materials

Supplementary materials-WB

Supplementary materials-RNAseq_result

## Data Availability

All data generated or analyzed during this study are included in this published article. The processed data are available from the corresponding author upon reasonable request.
