# Supplementary material for: NAT10 promotes gallbladder cancer progression by remodeling cholesterol metabolism via PCSK9 mRNA acetylation
Source: Cell Death Discov. 2026 Apr 16;12:251. doi: 10.1038/s41420-026-03104-z (PMC13201734; doi:10.1038/s41420-026-03104-z)
Supplement: Supplementary file 1 — Supplementary materials [file 41420_2026_3104_MOESM1_ESM.pdf]

Supplementary materials

Supplementary Table 1. siRNA and shRNA of genes

Gene	siRNA
NAT10-siRNA-1	GGCCAAAGCUGUCUUGAAA
NAT10-siRNA-2	CUGAUAACCUCCAUACUCU
PCSK9-siRNA-1	CCAAGAUCCUGCAUGUCUUTT
PCSK9-siRNA-2	GGUCACCGACUUCGAGAAUTT
NAT10-shRNA-1	CCGGCGGCCATCTCTCGCATCTATTCTCG
	AGAATAGATGCGAGAGATGGCCGTTTTTT
NAT10-shRNA-2	CCGGGAGATGTATTCACGGAATATGCTCG
	AGCATATTCCGTGAATACATCTCTTTTTT

Supplementary Table 2. Primer of genes

Gene	primer sequences
NAT10 forward primer	ATAGCAGCCACAAACATTCGC
NAT10 reverse primer	ACACACATGCCGAAGGTATTG
PCSK9 forward primer	CCTGGAGCGGATTACCCCT
PCSK9 reverse primer	CTGTATGCTGGTGTCTAGGAGA
SREBF2 forward primer	CTGCAACAACAGACGGTAATGA
SREBF2 reverse primer	CCATTGGCCGTTTGTGTCAG
HMGCS1 forward primer	CATTAGACCGCTGCTATTCTGTC
HMGCS1 reverse primer	TTCAGCAACATCCGAGCTAGA
HMGCR forward primer	TGATTGACCTTTCCAGAGCAAG
HMGCR reverse primer	CTAAAATTGCCATTCCACGAGC
MVK forward primer	CATGGCAAGGTAGCACTGG
MVK reverse primer	GATACCAATGTTGGGTAAGCTGA
MVD forward primer	GGACCGGATTTGGCTGAATG
MVD reverse primer	CCCATCCCGTGAGTTCCTC
IDI1 forward primer	TGGATAAAACCCCTGTGGTG
IDI1 reverse primer	CAACATCCGGCATAACTGTG
FDFT1 forward primer	GCAACGCAGTGTGCATATTTT
FDFT1 reverse primer	CGCCAGTCTGGTTGGTAAAGG
SQLE forward primer	CGCCAGTCTGGTTGGTAAAGG
SQLE reverse primer	CAGGGATACCCTTTAGCAGTTTT
GAPDH forward primer	GGAGCGAGATCCCTCCAAAAT
GAPDH reverse primer	GGCTGTTGTCATACTTCTCATGG

Supplementary Table 3. Information of antibody

Antibody	Catalogue	Dilution	Company
NAT10	66548	1:1000	Cell Signaling Technology, USA
PCSK9	85813	1:1000	Cell Signaling Technology, USA
SREBF2	A25685	1:1000	ABclonal Technology, China
HMGCR	A1633	1:2000	ABclonal Technology, China
FDFT1	A6229	1:1000	ABclonal Technology, China
LDLR	A20808	1:2000	ABclonal Technology, China
GAPDH	A19056	1:50000	ABclonal Technology, China
mTOR	A2445	1:2000	ABclonal Technology, China
p-mTOR	AP0115	1:1000	ABclonal Technology, China
AKT	A18675	1:2000	ABclonal Technology, China
p-AKT	AP1208	1:1000	ABclonal Technology, China
PI3K	A27717	1:2000	ABclonal Technology, China
H3	A22348	1:10000	ABclonal Technology, China

Supplementary Figure1.

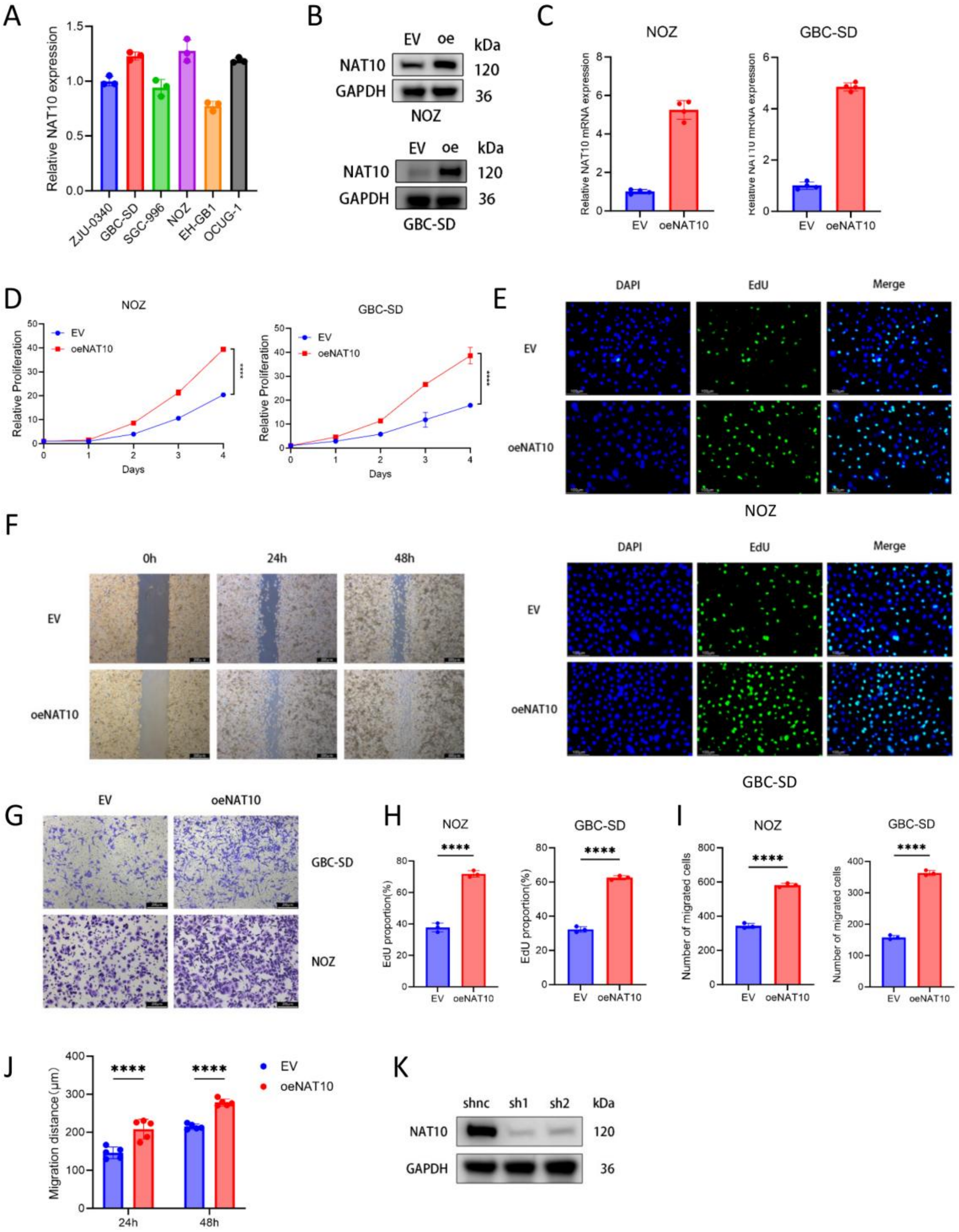

A Basal mRNA expression of NAT10 across a panel of GBC cell lines. **B** Western blot analysis confirming NAT10 overexpression efficiency. **C** Relative PCSK9 mRNA levels upon NAT10 overexpression. **D** Cell proliferation assessed by CCK-8 assay following NAT10 overexpression. **E, F** Proliferation assessed by EdU assay (E) and migration assessed by wound

healing assay (F) in a representative GBC cell line after NAT10 overexpression. Scale bars: 100 μm. **G, H** Proliferation assessed by EdU assay (G) and migration assessed by Transwell assay (H) in another GBC cell line after NAT10 overexpression. Scale bars: 100 μm (G) and 200 μm (H). **I** Western blot analysis confirming NAT10 knockdown efficiency by shRNA.

Data are presented as mean ± SD. *$p < 0.05$, **$p < 0.01$, ***$p < 0.001$, ****$p < 0.0001$.

Supplementary Figure 2.

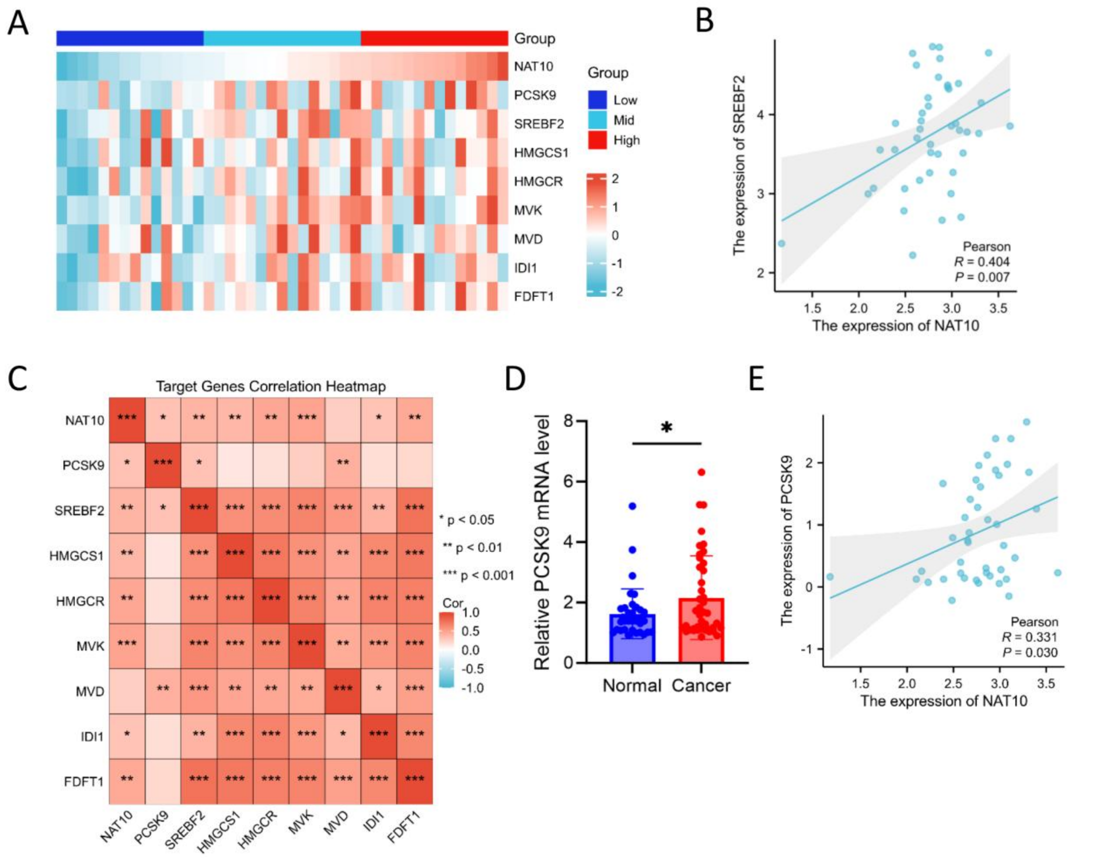

A Heatmap showing expression levels of key cholesterol metabolism genes (PCSK9, SREBF2, HMGCS1, HMGCR, MVK, MVD, IDI1, FDFT1) in tumors stratified by high, medium, and low NAT10 expression. **B** Correlation between NAT10 and SREBF2 expression. **C** Correlation between NAT10 and the panel of cholesterol metabolism genes. **D** PCSK9 expression in GBC tumors versus adjacent normal tissues. **E** Correlation between NAT10 and PCSK9 expression. Data are presented as mean ± SD. *p < 0.05, **p < 0.01, ***p < 0.001, ****p < 0.0001.

Supplementary Figure 3.

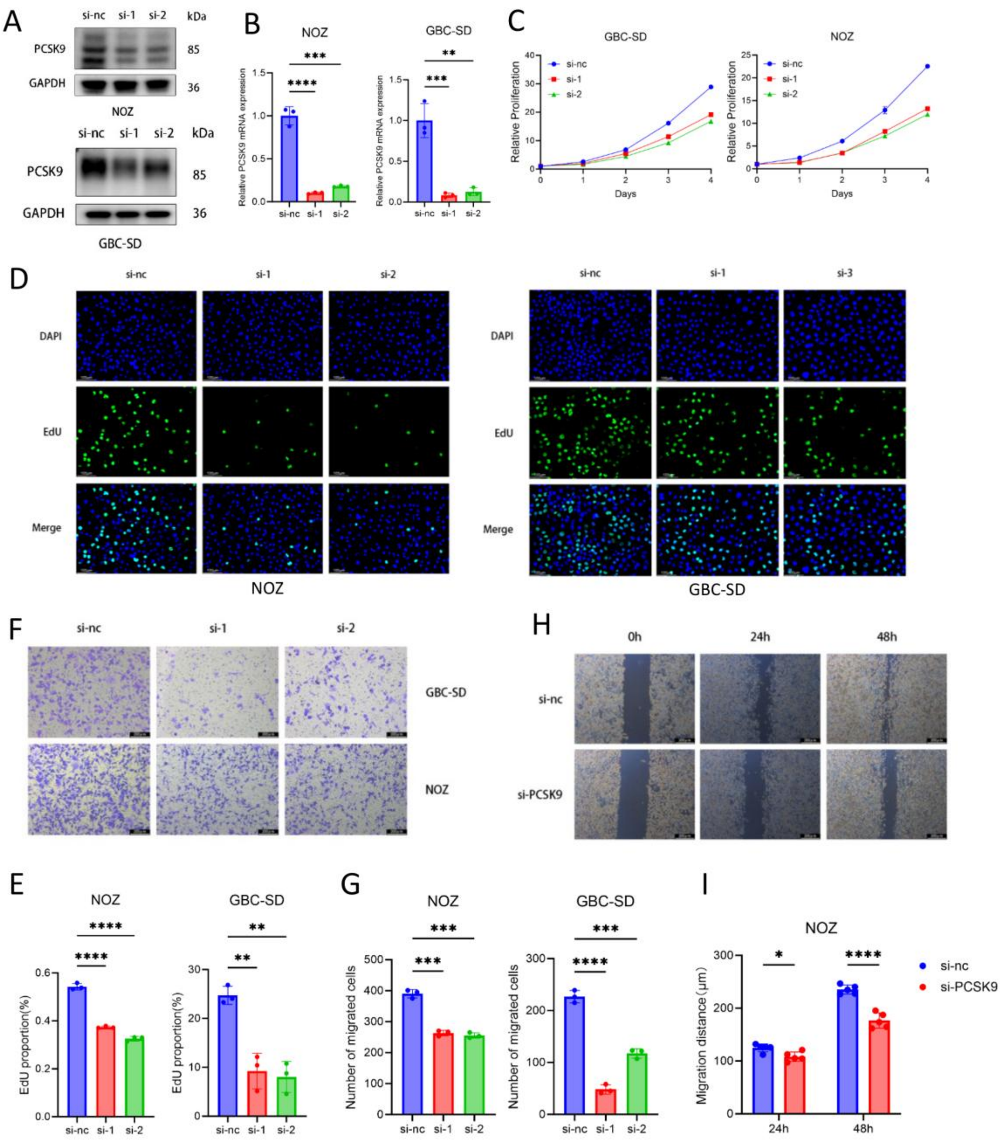

A Western blot analysis confirming PCSK9 knockdown efficiency. **B** Relative PCSK9 mRNA levels upon PCSK9 knockdown. **C** Cell proliferation assessed by CCK-8 assay after PCSK9 knockdown. **D, E** Proliferation assessed by EdU assay after PCSK9 knockdown. Scale bars: 100 μm. **F, G** Cell migration assessed by Transwell assay after PCSK9 knockdown. Scale bars: 200 μm. **H, I** Cell migration assessed by wound healing assay after PCSK9 knockdown. Scale bars: 100 μm. Data are presented as mean ± SD. $*p < 0.05$, $**p < 0.01$, $***p < 0.001$, $****p < 0.0001$.

Supplementary Figure 4.

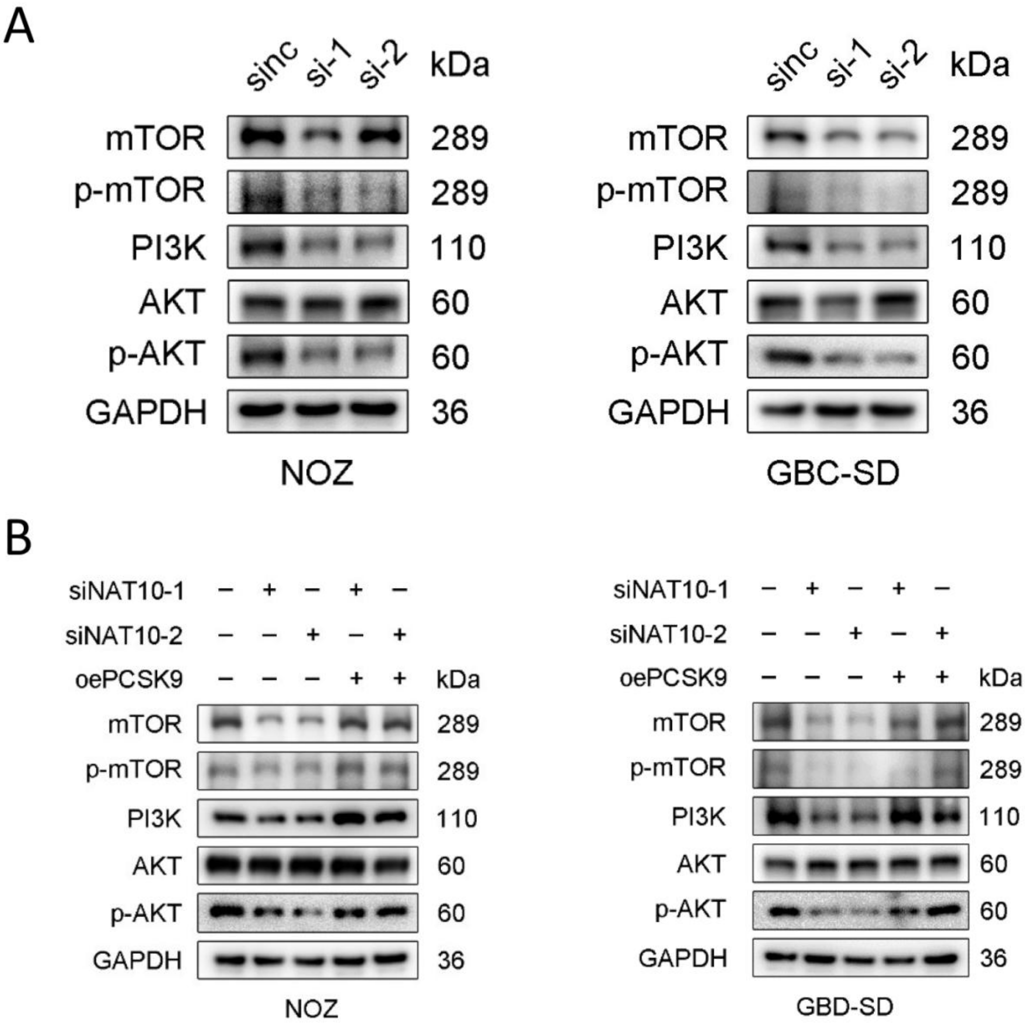

A Western blot analysis of key PI3K/AKT/mTOR pathway proteins (PI3K, AKT, p-AKT, mTOR, p-mTOR) in NOZ and GBC-SD cells after PCSK9 knockdown. GAPDH served as a loading control. **B** Western blot analysis of PI3K/AKT/mTOR pathway proteins after restoration of PCSK9 expression in NAT10-knockdown cells.

Supplementary Figure 5.

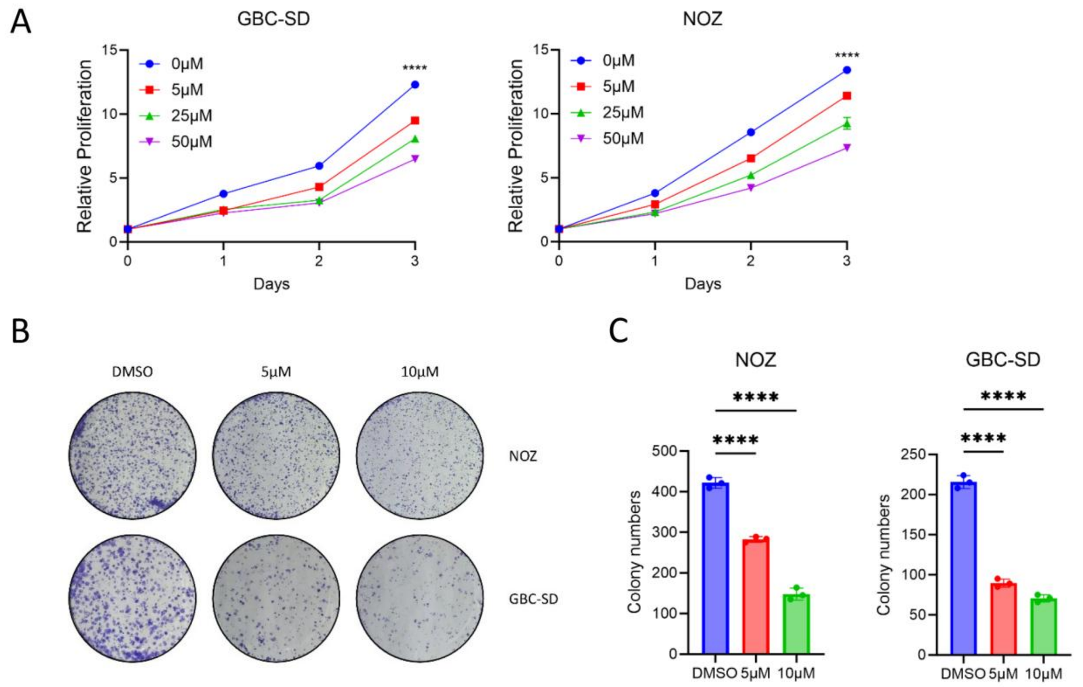

A Cell proliferation measured by CCK-8 assay after treatment with PCSK9-IN-11 at indicated concentrations (0, 5, 25, 50 μM). **B, C** Clonogenic ability assessed by colony formation assay after treatment with PCSK9-IN-11 (0, 5, 10 μM). Data are presented as mean ± SD. *p < 0.05, **p < 0.01, ***p < 0.001, ****p < 0.0001.

Supplementary Figure 6.

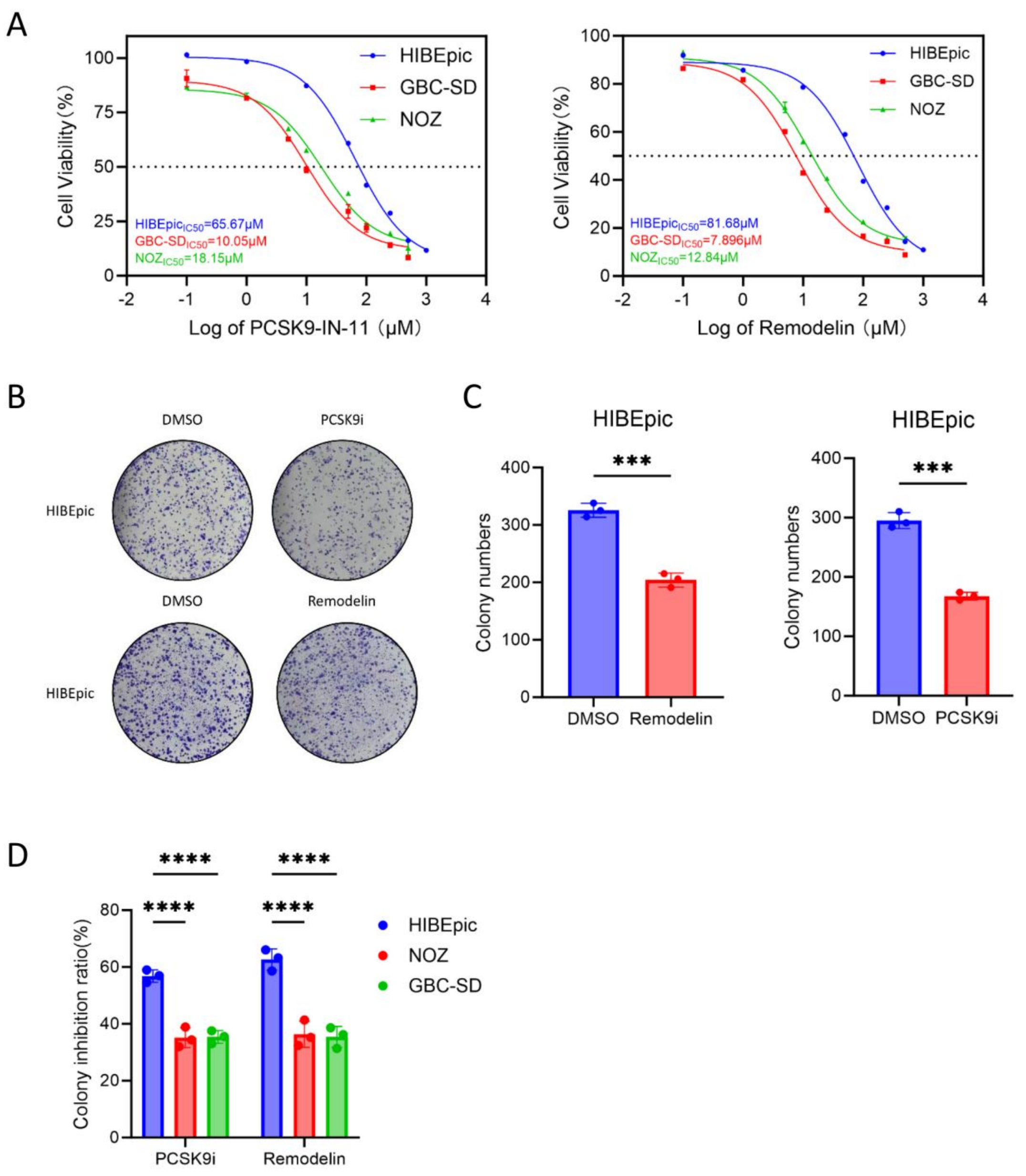

A Dose-response curves and IC_{50} values of Remodelin and PCSK9-IN-11 in HIBEpic, GBC-SD, and NOZ. **B, C** Representative images from clonogenic assays of HIBEpic treated with Remodelin (10 μM) or PCSK9-IN-11 (10 μM). **D** Quantitative analysis of clonogenic inhibition rates for both compounds across the three cell lines. Data are presented as mean ± SD. $^*p < 0.05$, $^{**}p < 0.01$, $^{***}p < 0.001$, $^{****}p < 0.0001$.
