# Supplementary material for: NAT10 promotes gallbladder cancer progression by remodeling cholesterol metabolism via PCSK9 mRNA acetylation
Source: Cell Death Discov. 2026 Apr 16;12:251. doi: 10.1038/s41420-026-03104-z (PMC13201734; doi:10.1038/s41420-026-03104-z)

1. Figure2A

NAT10

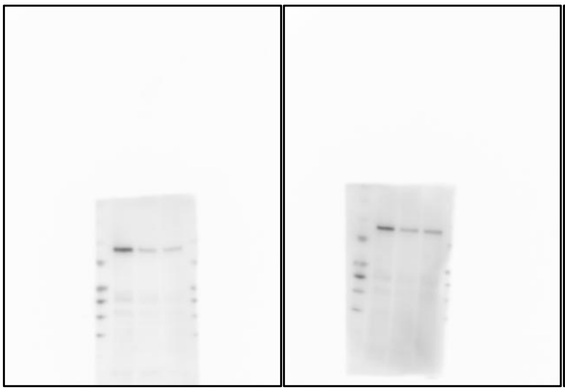

GAPDH

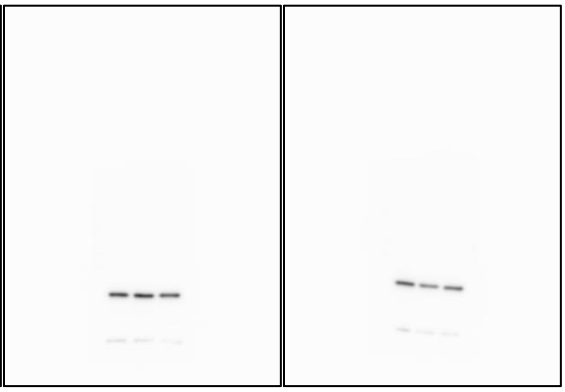

2. Figure3H

NAT10

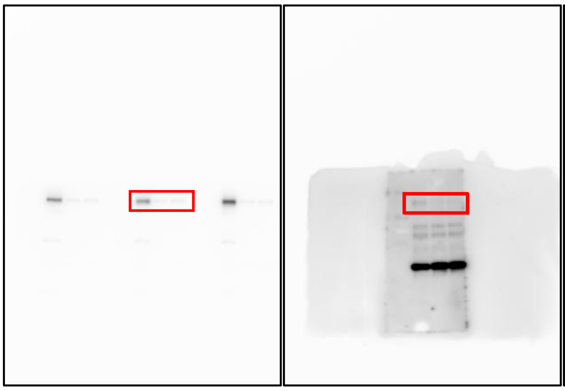

mTOR

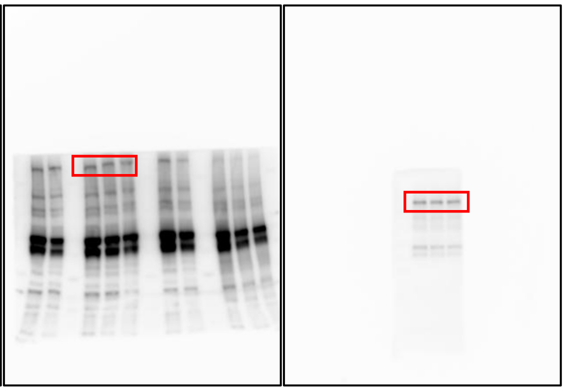

p-mTOR

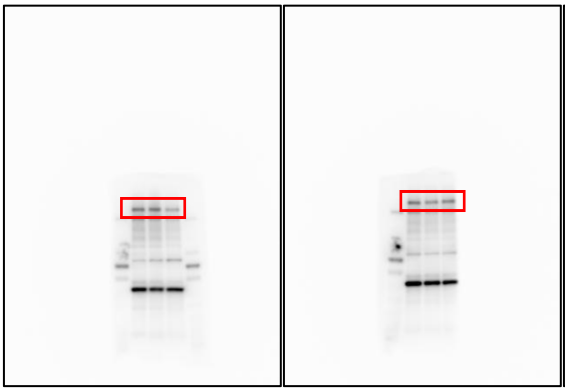

AKT

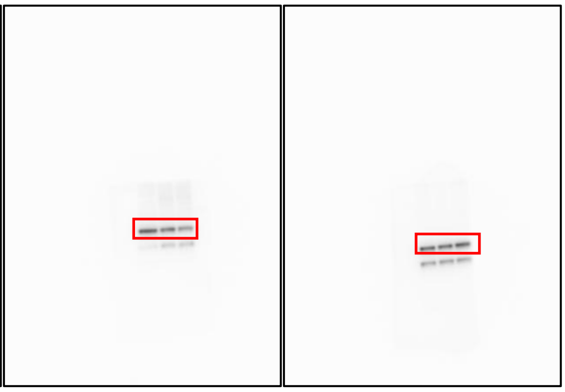

p-AKT

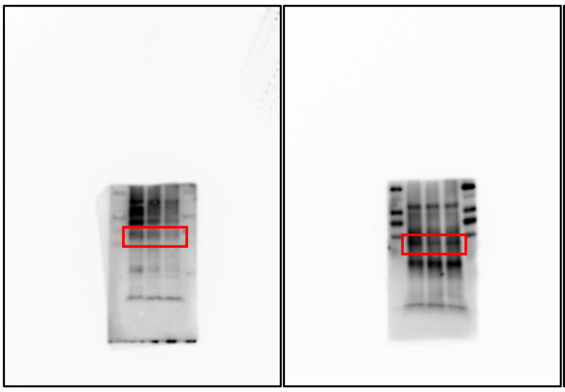

PI3K

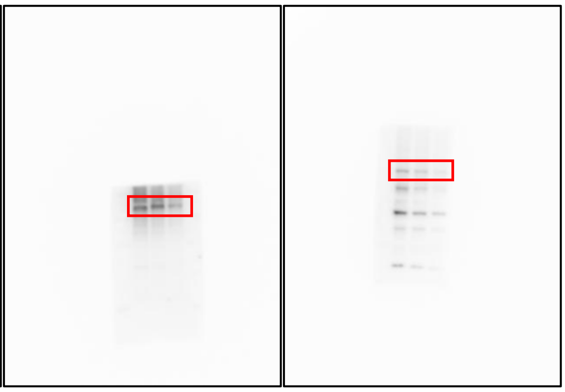

GAPDH

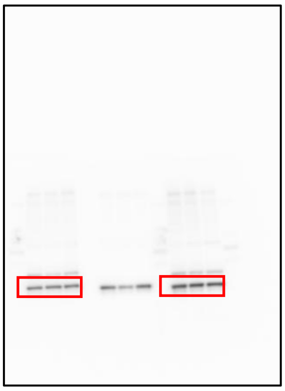

3. Figure4D

NAT10

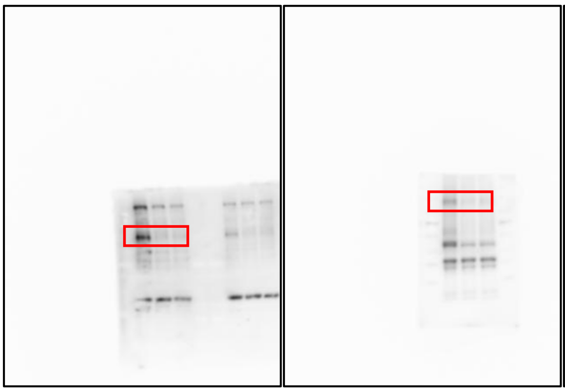

LDLR

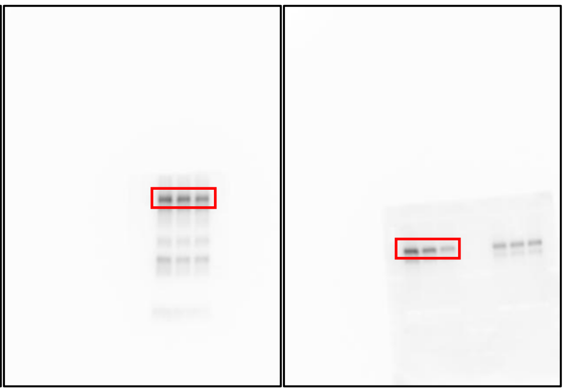

SREBF2

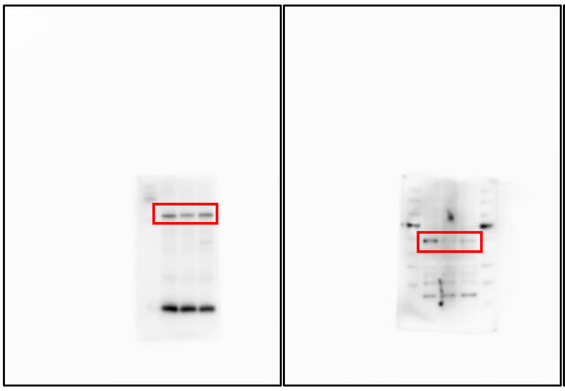

HMGCR

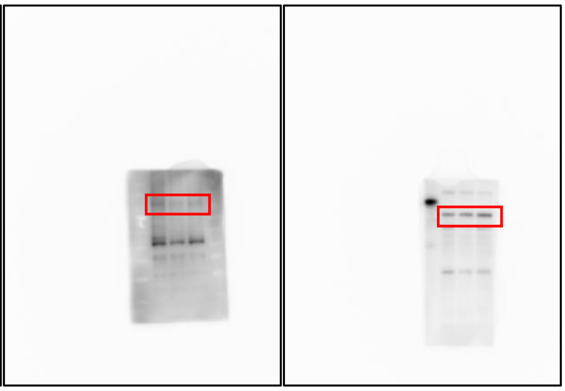

FDFT1

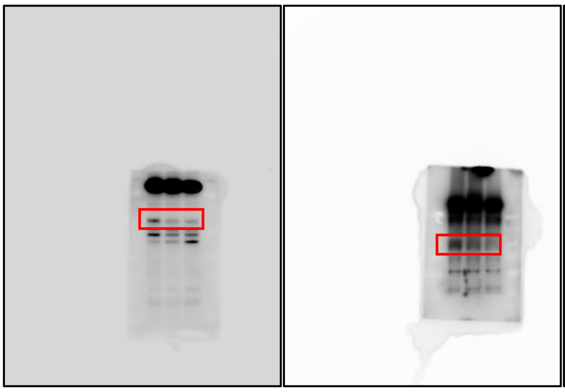

GAPDH

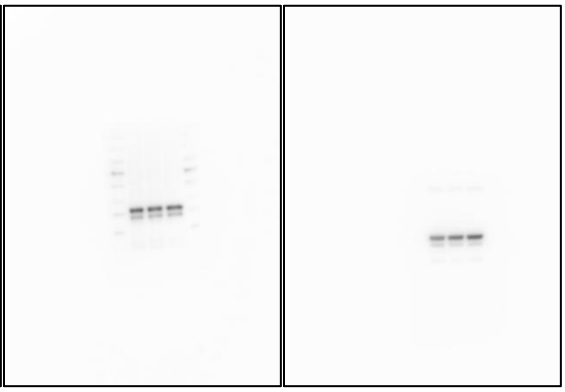

4. Figure4H

SREBF2 H3

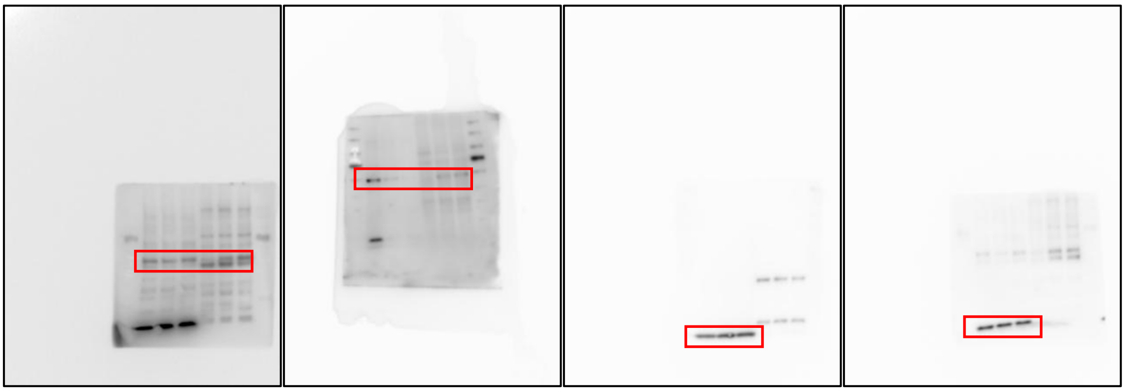

GAPDH

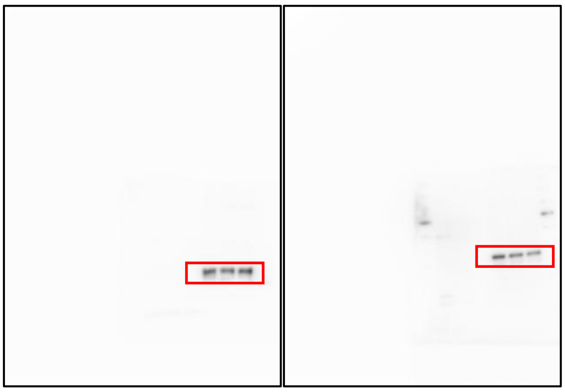

5. Figure5B

NAT10 PCSK9

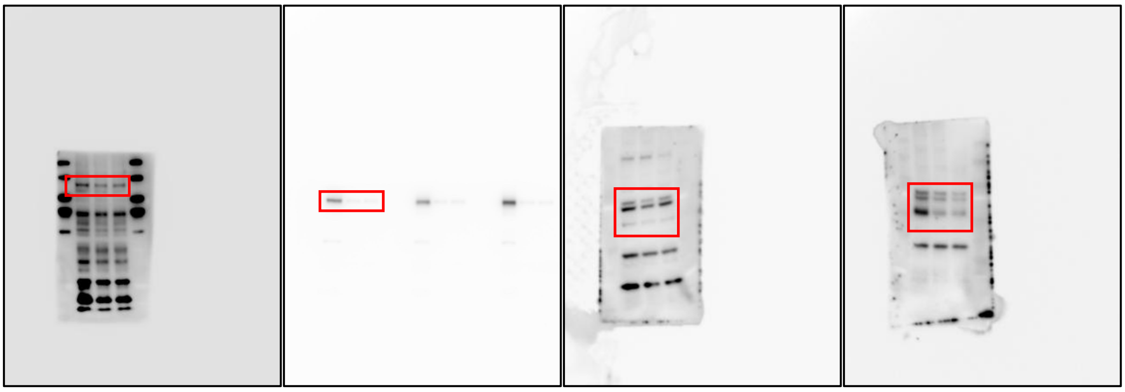

GAPDH

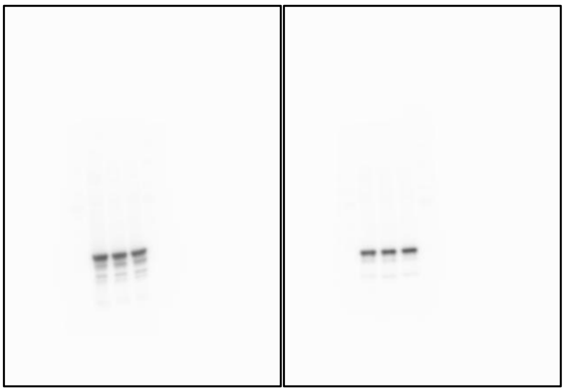

6. Figure5E

PCSK9

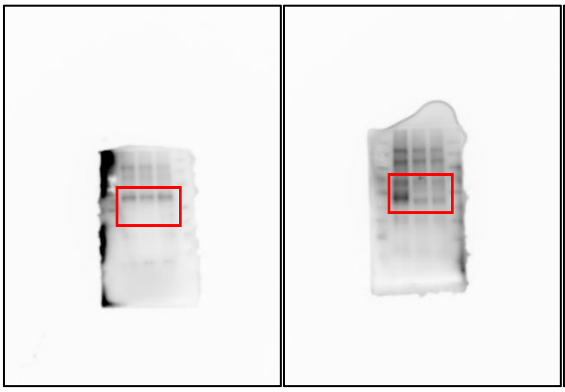

LDLR

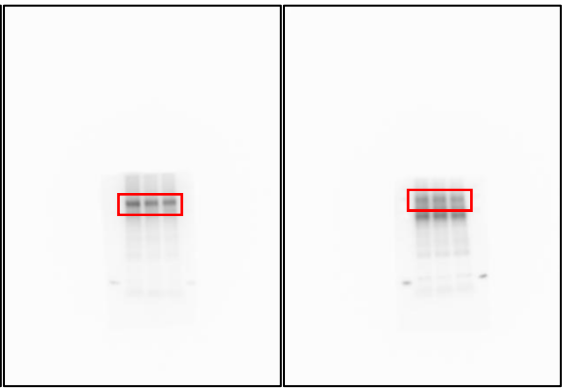

SREBF2

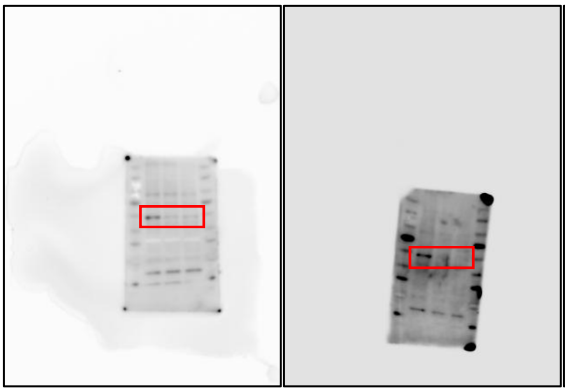

HMGCR

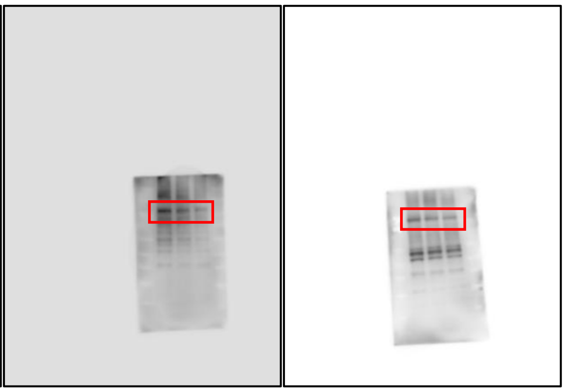

FDFT1

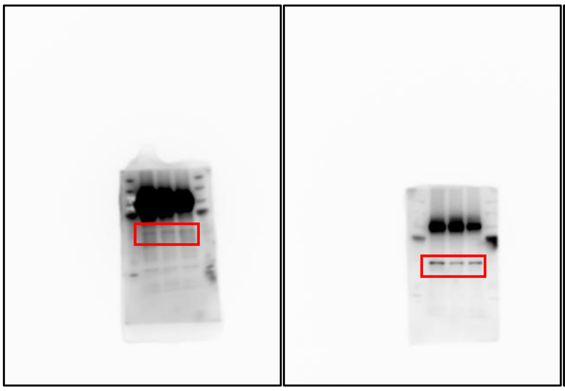

GAPDH

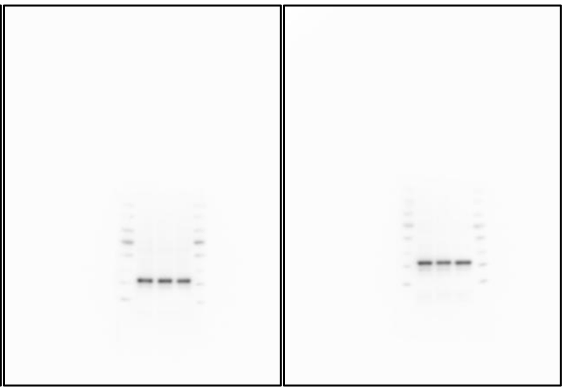

7. Figure5H

NAT10

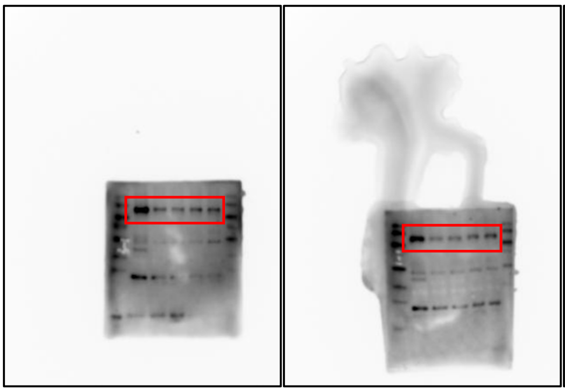

PCSK9

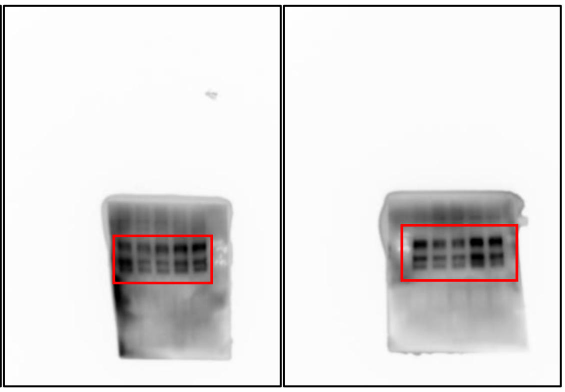

LDLR

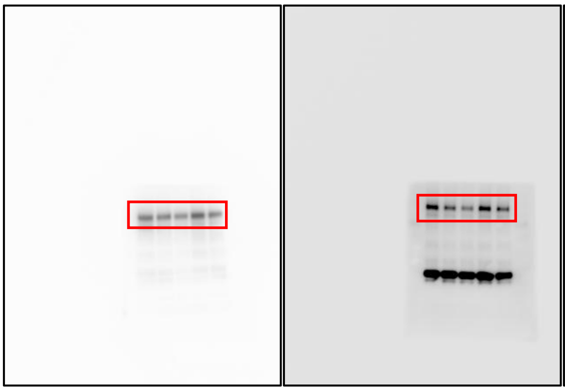

SREBF2

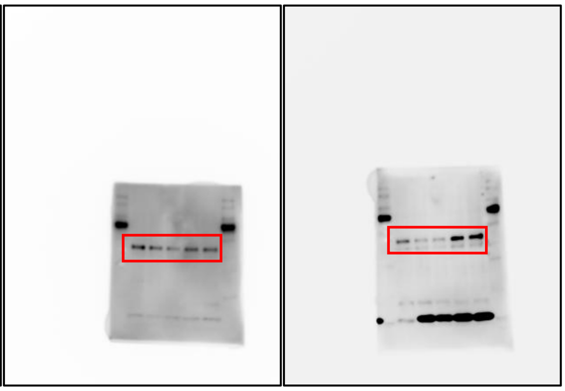

HMGCR

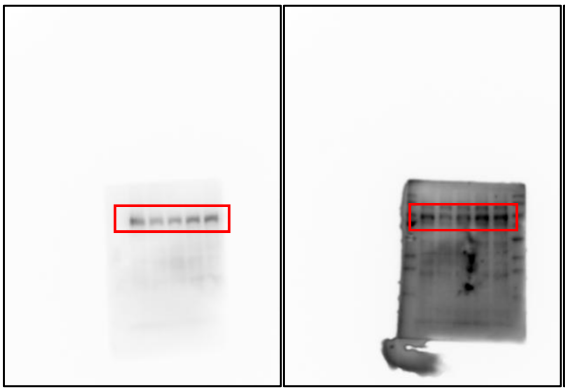

FDFT1

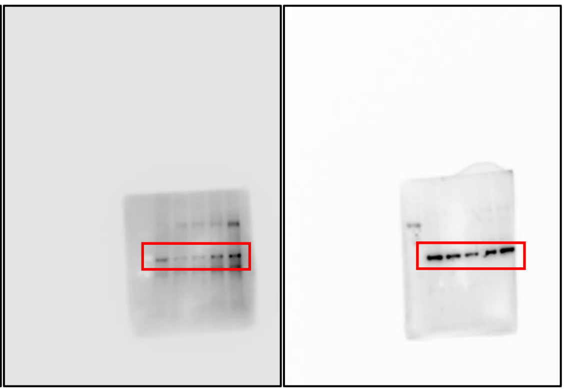

GAPDH

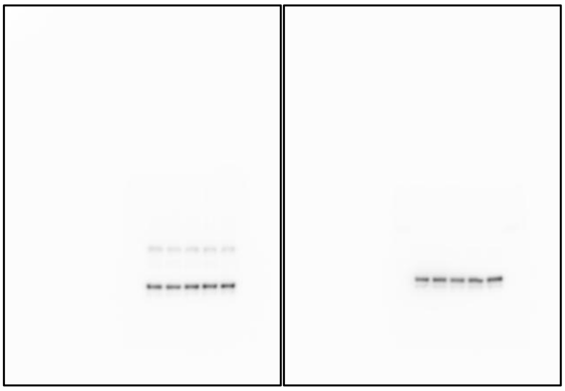

8. Figure5I

PCSK9

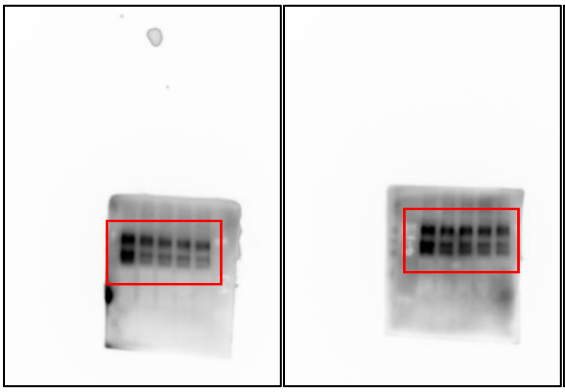

SREBF2

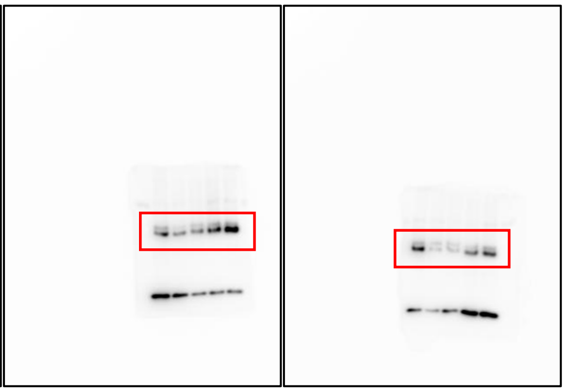

LDLR

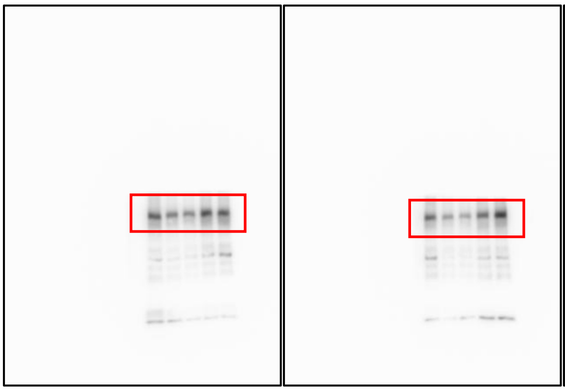

HMGCR

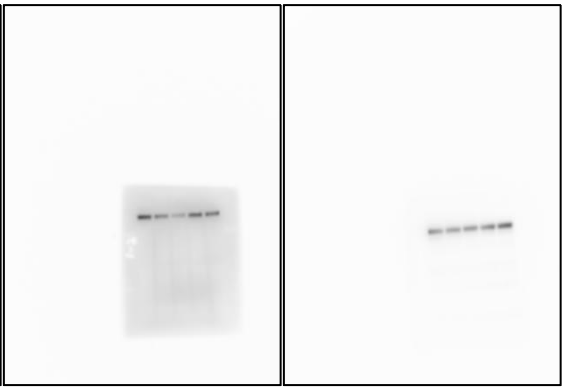

FDFT1

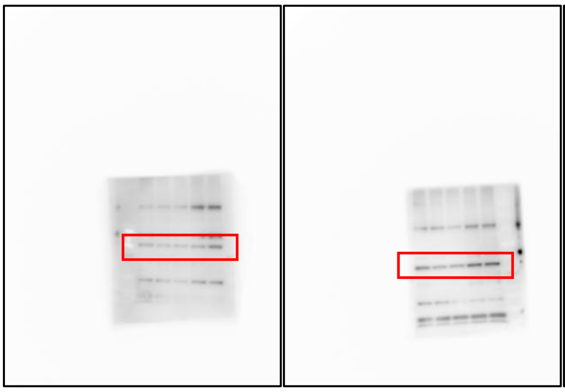

GAPDH

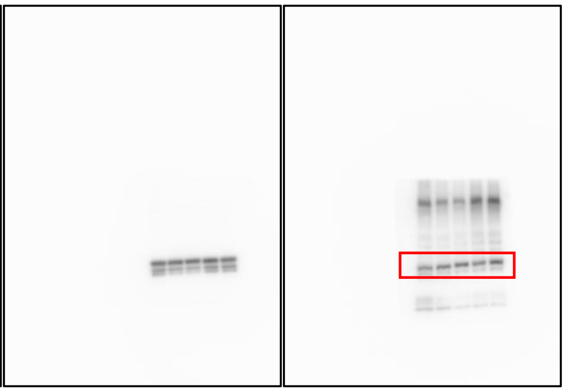

9. Figure6G

PCSK9

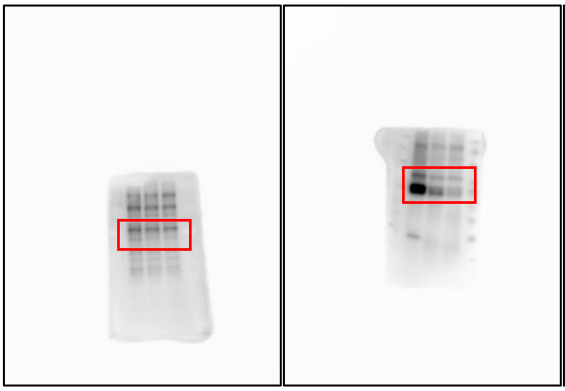

GAPDH

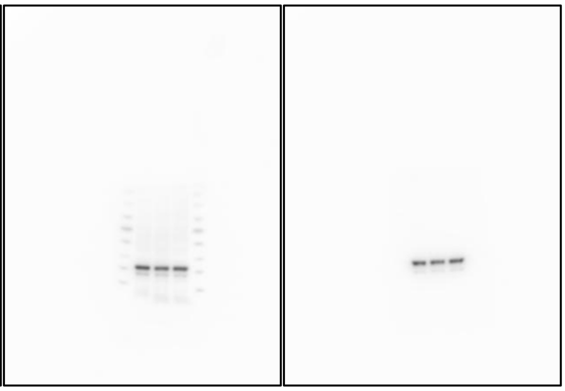

10. Figure6I

PCSK9

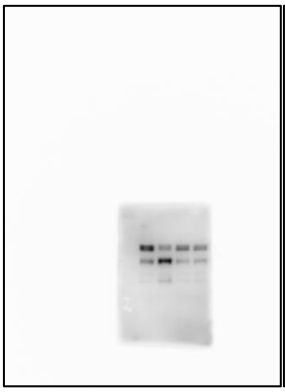

NAT10

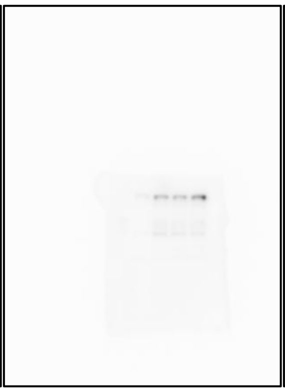

GAPDH

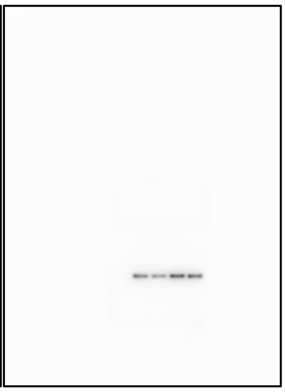

11. Figure7E

mTOR p-mTOR

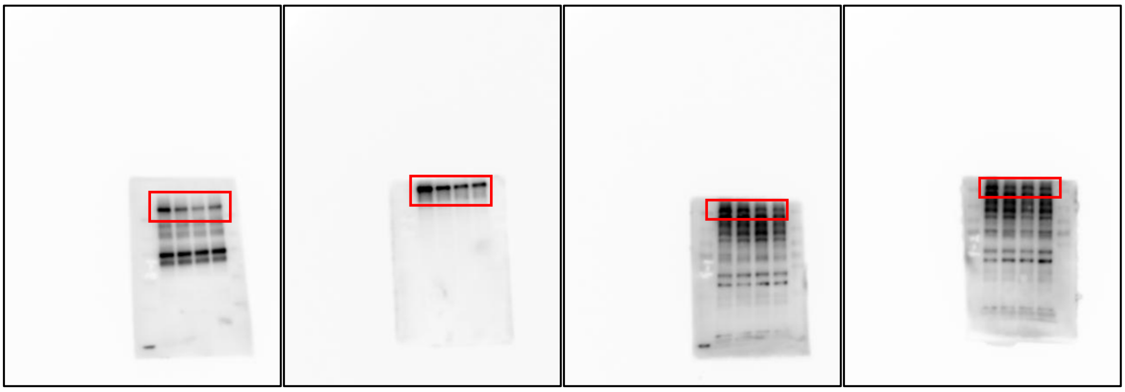

AKT p-AKT

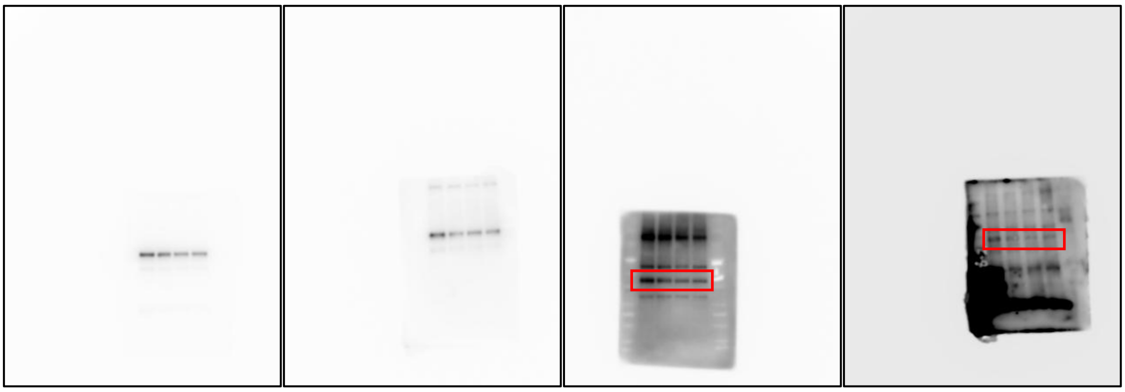

PI3K GAPDH

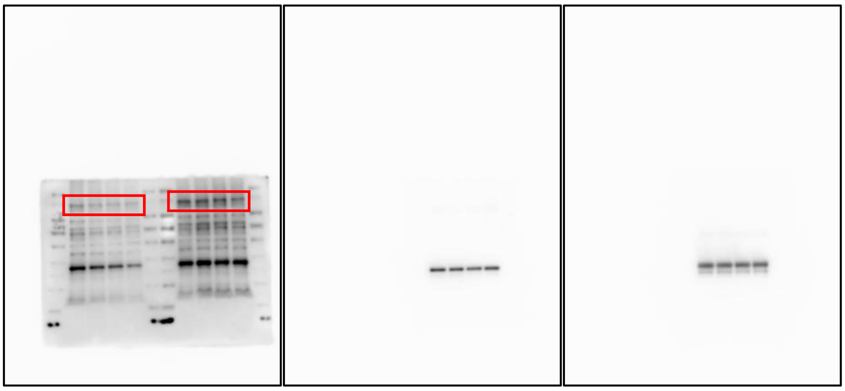

12. Figure7I

mTOR p-mTOR

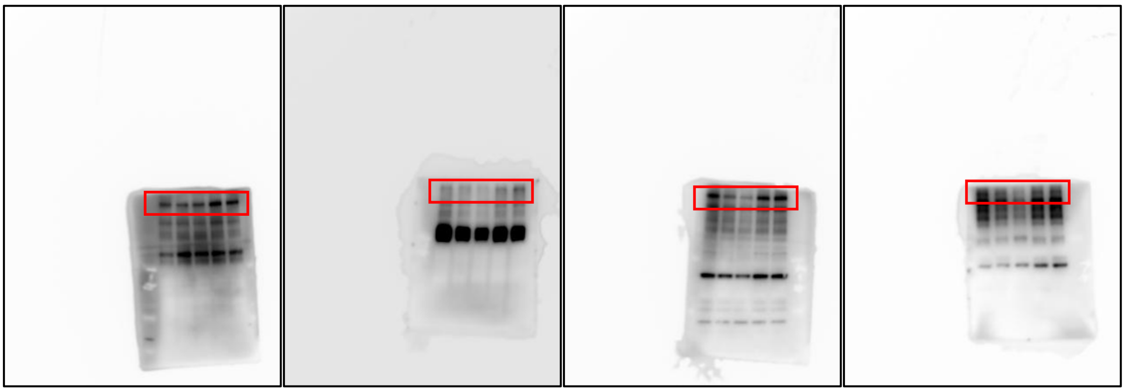

AKT p-AKT

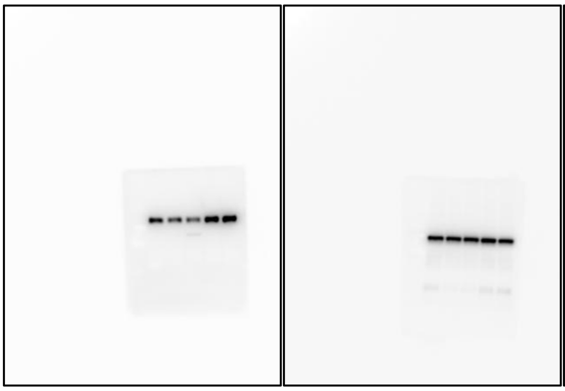
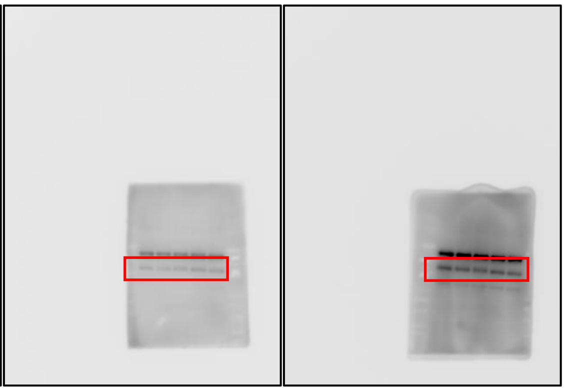

PI3K GAPDH

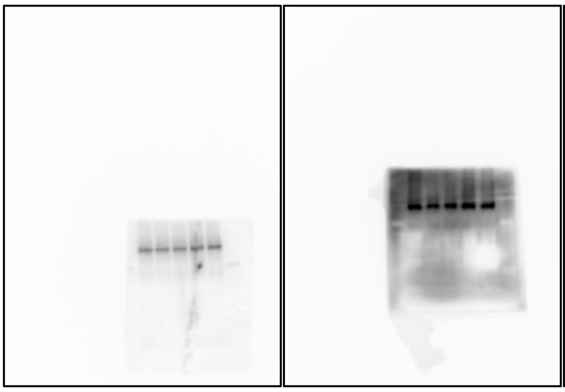
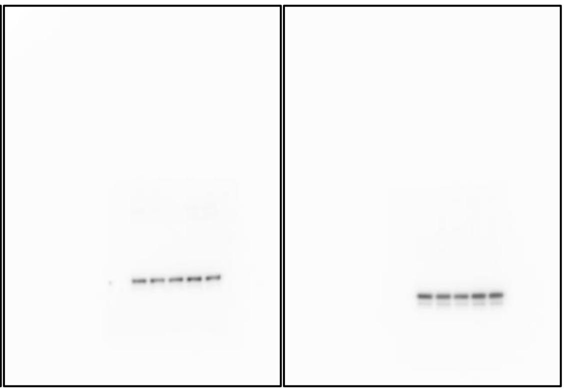

13. Supplementary Figure1A

NAT10 GAPDH

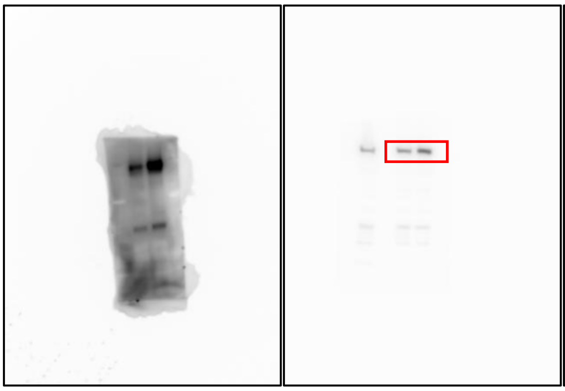
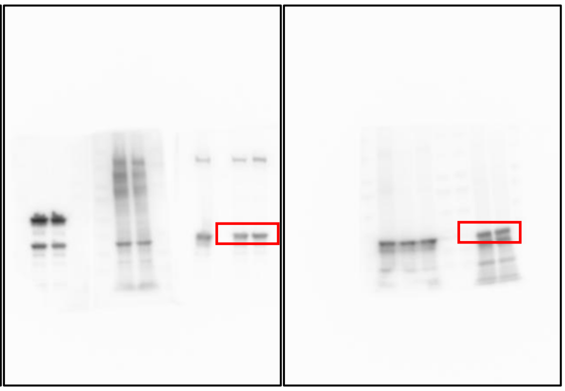

14. Supplementary Figure1K

NAT10 GAPDH

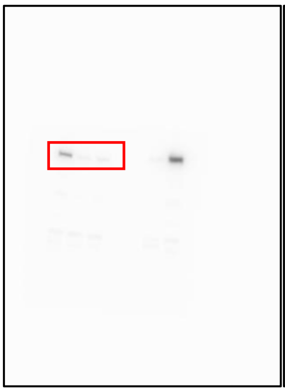
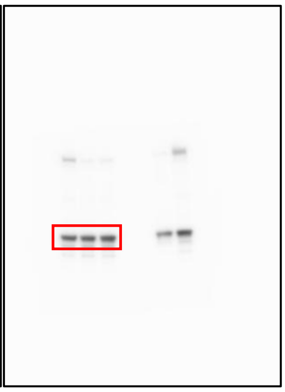

15. Supplementary Figure3A

PCSK9 GAPDH

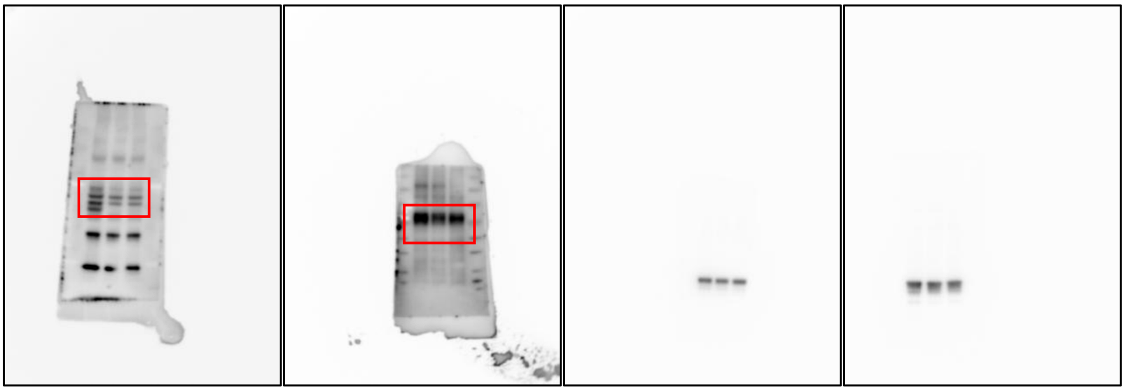

16. Supplementary Figure4A

mTOR p-mTOR PI3K

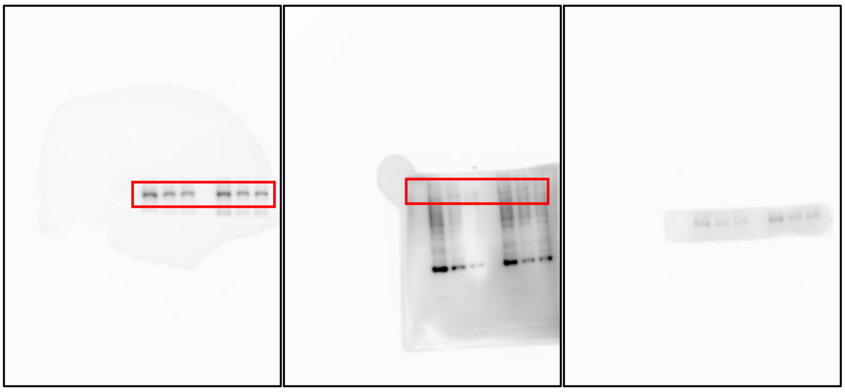

AKT p-AKT

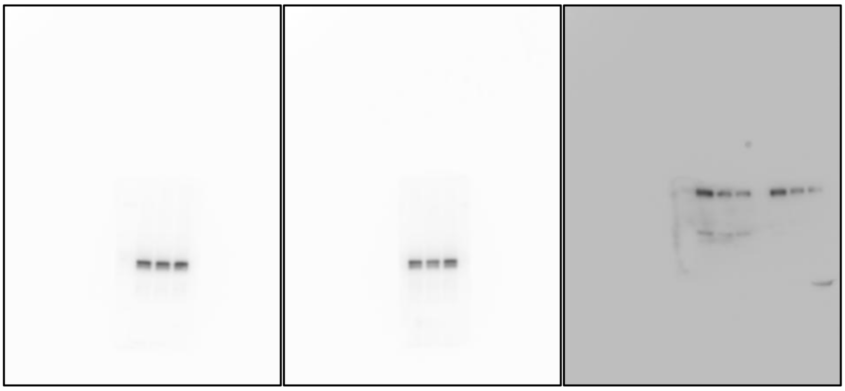

GAPDH

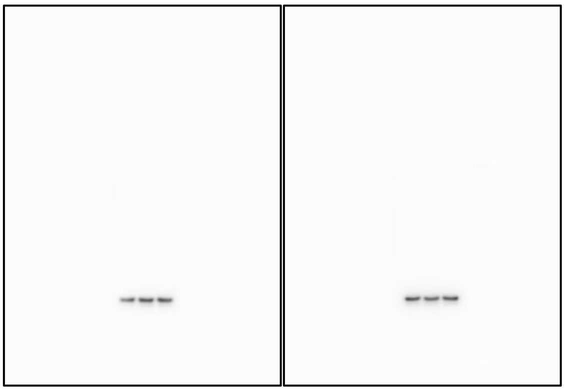

17. Supplementary Figure4B

mTOR

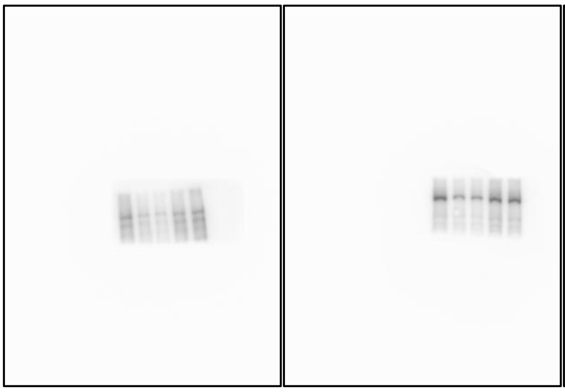

p-mTOR

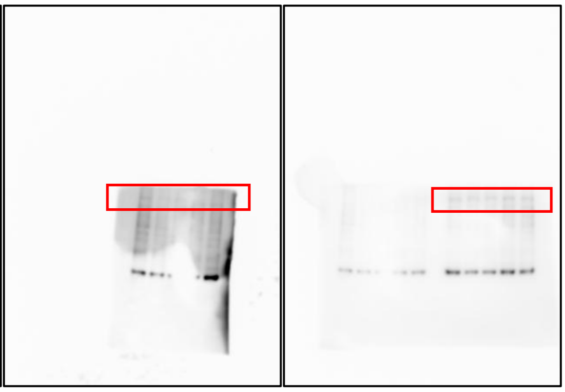

PI3K

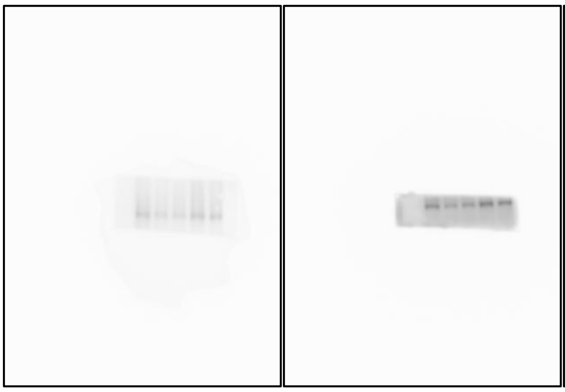

AKT

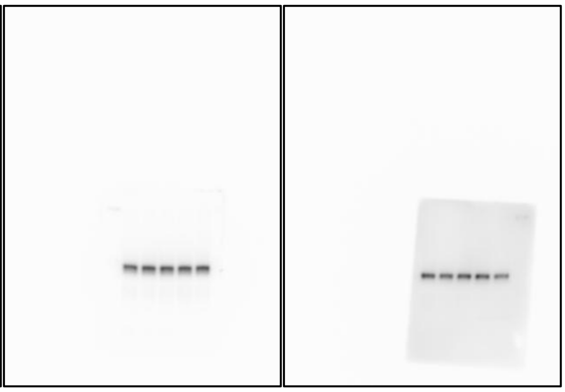

p-AKT

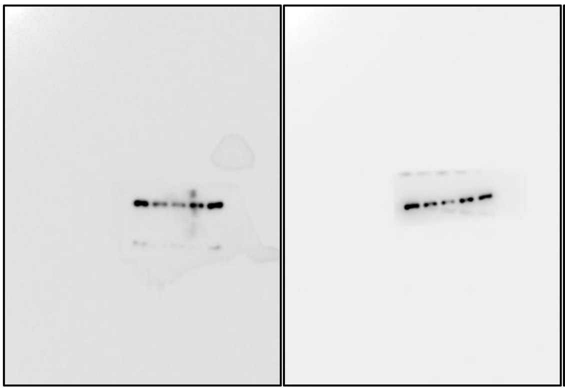

GAPDH

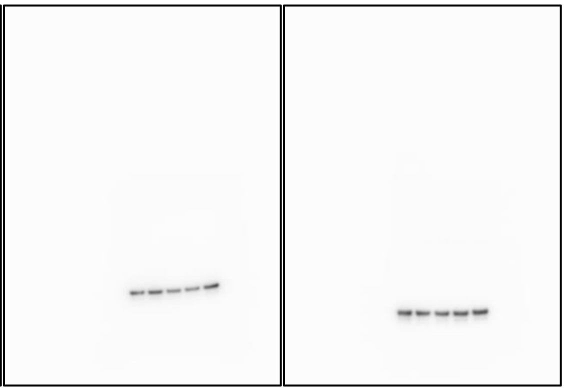

Supplement: Supplementary file 2 — Supplementary materials-WB [file 41420_2026_3104_MOESM2_ESM.pdf]
